# Richness, systematics, and distribution of molluscs associated with the macroalga *Gigartina skottsbergii* in the Strait of Magellan, Chile: A biogeographic affinity study

**DOI:** 10.3897/zookeys.519.9676

**Published:** 2015-08-31

**Authors:** Sebastián Rosenfeld, Cristian Aldea, Andrés Mansilla, Johanna Marambio, Jaime Ojeda

**Affiliations:** 1 Laboratorio de Macroalgas Antárticas y Subantárticas, Universidad de Magallanes, Casilla 113-D, Punta Arenas, Chile; 2 Laboratorio de Ecología y Medio Ambiente, Instituto de la Patagonia, Universidad de Magallanes; 3 Programa GAIA-Antártica, Universidad de Magallanes; 4 Instituto de Ecología y Biodiversidad (IEB), Santiago; 5 Parque Etnobotánico Omora, Sede Puerto Williams, Universidad de Magallanes

**Keywords:** Mollusca, biodiversity, biogeography, algae beds, Magellan Region

## Abstract

Knowledge about the marine malacofauna in the Magellan Region has been gained from many scientific expeditions that were carried out during the 19th century. However, despite the information that exists about molluscs in the Magellan Region, there is a lack of studies about assemblages of molluscs co-occurring with macroalgae, especially commercially exploitable algae such as *Gigartina skottsbergii*, a species that currently represents the largest portion of carrageenans within the Chilean industry. The objective of this study is to inform about the richness, systematics, and distribution of the species of molluscs associated with natural beds in the Strait of Magellan. A total of 120 samples from quadrates of 0.25 m^2^ were obtained by SCUBA diving at two sites within the Strait of Magellan. Sampling occurred seasonally between autumn 2010 and summer 2011: 15 quadrates were collected at each site and season. A total of 852 individuals, corresponding to 42 species of molluscs belonging to Polyplacophora (9 species), Gastropoda (24), and Bivalvia (9), were identified. The species richness recorded represents a value above the average richness of those reported in studies carried out in the last 40 years in sublittoral bottoms of the Strait of Magellan. The biogeographic affinity indicates that the majority of those species (38%) present an endemic Magellanic distribution, while the rest have a wide distribution in the Magellanic-Pacific, Magellanic-Atlantic, and Magellanic-Southern Ocean. The molluscs from the Magellan Region serve as study models for biogeographic relationships that can explain long-reaching patterns and are meaningful in evaluating possible ecosystemic changes generated by natural causes or related to human activities.

## Introduction

In the South-eastern Pacific Ocean, the Magellanic biogeographic province (43°S to 56°S) is constituted by a large extension of channels and fjords with diverse coastal environments from glacial influence to direct exposure by the Pacific Ocean (Camus 2001, Spalding et al. 2007). Two biogeographic districts have been categorised for this biogeographic province: the Austral and the Subantarctic. The latter extends from about 52°–53°S to 56°S (Camus 2001); in other words, from the Strait of Magellan to Cape Horn. This territory is characterised by different environmental conditions that determine sub-areas of physiogeology and orography, geology, soils, and differentiated climates (Pisano 1977). Within the Subantarctic biogeographic region, the Strait of Magellan connects the Pacific and Atlantic oceans. For this reason, the Strait of Magellan offers unique characteristics for studying biodiversity and, specifically, aspects related to biogeography (Ríos et al. 2003).

Knowledge about a large part of the marine fauna in the Magellan Region was first attained from scientific expeditions carried out during the 19th century. The historical contributions to the knowledge of molluscs from the Magellanic biogeographic province have been detailed by Reid and Osorio (2000), Cárdenas et al. (2008), and Aldea and Rosenfeld (2011). Currently, many researchers have contributed to the knowledge about these molluscs, principally in descriptive taxonomy and ecology in the Magellan Region (e.g. Linse 2002, Ríos et al. 2003, Zelaya and Ituarte 2003, 2004, Pastorino 2005a, 2005b, Linse et al. 2006, Schwabe et al. 2006, Sirenko 2006, Zelaya and Geiger 2007, Ojeda et al. 2010, Aldea and Rosenfeld 2011, Rosenfeld and Aldea 2011, Rosenfeld et al. 2011, Signorelli and Pastorino 2011). Recently, new contributions have been made using molecular tools in order to study specific groups of molluscs (e.g. Espoz et al. 2004, Aranzamendi et al. 2009, Gonzalez-Wevar et al. 2010, 2011). One crucial aspect of molluscs from these latitudes is their biogeographic relationship that can explain “long reaching patterns” (e.g. Linse et al. 2006, Clarke et al. 2007, Fortes and Absalao 2011). Therefore, molluscs are interesting as a study group to evaluate possible ecosystemic changes generated by natural or human causes.

Although much knowledge exists about molluscs from the Magellan Region, the majority of this knowledge has been centred only on the characterisation of the taxon and not on the search for assemblages and biogeographic patterns. Some contributions to this interaction have come from studies on invertebrates associated with giant kelp, *Macrocystis pyrifera* (Ojeda and Santelices 1984, Adami and Gordillo 1999, Rios et al. 2007). Currently, the only macroalgae in the Magellan Region with massive commercial exploitation corresponds to the carregeanofite *Gigartina skottsbergii*. This species is distributed from 39°52'S (Romo et al. 2001) toward the Antarctic Peninsula (Wiencke and Clayton 2002). *Gigartina skottsbergii* is characterised as forming a dense sublittoral bed, reaching a biomass density of around 1773 g/m^2^ and densities of 15 individuals/m^2^ (Ávila et al. 2004). The extraction of this species has the objective of providing the principal raw material for the production of carrageenan hydrocolloid (carrageenan), a gel with multiple applications in the food and cosmetics industries (Romo et al. 2001; Pujol et al. 2006; Barahona et al. 2012). Due to the growing national and international demand for this raw material, algae beds have suffered significant losses and their restoration has been quite slow, showing largely damaged communities in beds of Puerto Montt (~41°S; Romo et al. 2001). For this reason, a good share of the extractive pressure has moved toward the south, especially in the area of the Gulf of Penas (~47°S) as well as the Magellan Region (~53°S; Romo et al. 2001, Mansilla et al. 2008).

Differing from other distribution sites of *Gigartina skottsbergii*, the Strait of Magellan still has a natural bed of *Gigartina skottsbergii* (Ávila et al. 2004), and it is important for analysis for two reasons: i) describing the current situation of the fauna present in natural beds and ii) because analysis of the systematics and distribution of molluscs throughout the Strait of Magellan is a good model to characterise possible faunistic connections between different environments (e.g. Atlantic-Pacific). Thus, populations of *Gigartina skottsbergii* in the Magellan Region constitute an excellent alternative to study the benthic biodiversity. Here it is possible to study molluscs that are associated with algae and form beds that provide a shelter for associated species (Mansilla 2013), potentially contribute to conservation (Gray 1997, Fernández et al. 2000, Lancellotti and Vásquez 2000) or allow to determine an eventual loss of diversity for the function of the ecosystem (Purvis and Hector 2000). In this sense, the objective of this study is to describe the species richness and distribution of the mollusc species associated with the natural bed of *Gigartina skottsbergii* in the Strait of Magellan, and to evaluate the biogeographic affinities of all the species.

## Material and methods

**Figure 1.**
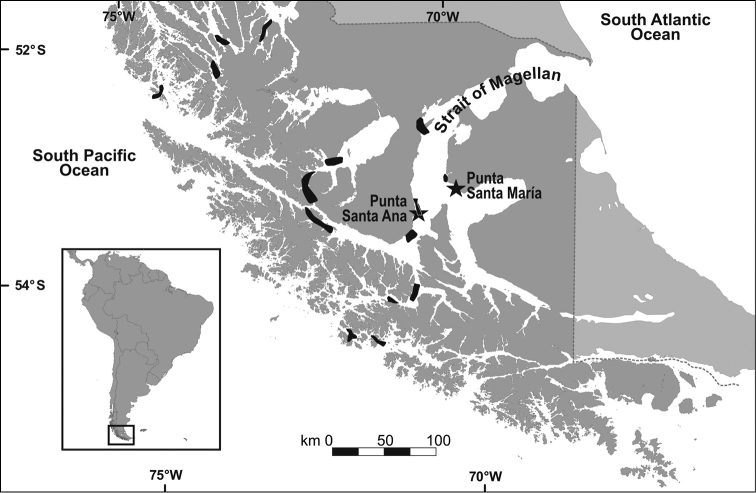
Study area. Location of sampling sites: Punta Santa Ana and Punta Santa María (stars) and natural beds of *Gigartina skottsbergii* (shading areas, extrated from Ávila et al. 2004).

The study area was localised in two sampling sites with the presence of a bed of *Gigartina skottsbergii* in the Strait of Magellan: i) Punta Santa Maria, located in Tierra del Fuego (53°21'S – 70°27'W), and ii) Punta Santa Ana, located 60 km to the south of Punta Arenas (53°37'S – 70°52'W) (Fig. 1). The samples were obtained by SCUBA diving at ~10 m depth in quadrates of 0.25m^2^, which were selected randomly within the bed. In each quadrate, all molluscs were collected, and also the substrate, where *Gigartina skottsbergii* settled, was investigated. Subsequently, the rocks were scraped to ensure that all the species and specimens were collected. Fifteen quadrates were sampled during one dive in each site and season, resulting in 60 quadrates per site (2 sites × 4 seasons × 15 quadrates). Sampling was carried out in autumn, winter, and spring of 2010, and in summer of 2011. The samples obtained were deposited in plastic bags, tagged and preserved in Formalin, diluted to 4–5% in seawater, and buffered with sodium borate.

### Systematics analysis

Taxonomic identification of the molluscs and the registry of the geographic distribution of each species was based on a complete study of the current literature (e.g. Reid and Osorio 2000, Linse 2002), as well as on classic works (e.g. Smith 1881, Rochebrune and Mabille 1889, Strebel 1905a, 1905b), systematics studies about specific taxa (e.g. Villaroel and Stuardo 1998, Pastorino and Harasewych 2000, Zelaya 2004), and academic databases available on the internet (Morris and Rosenberg 2005, USNM 2010).

All of the morphotypes that were identified at species level are included in this report, with the following information presented for each one: a) material examined, b) synonymy, c) remarks, and d) distribution. The material examined is detailed for each bed, showing the number of live specimens collected (spm.) and including the dimensions of the largest and smallest specimens. The synonymy is derived from the last taxonomic study. In the remarks, taxonomic, morphological, and/or ecological aspects are discussed. The distribution shows all previous records of the species, arranged from north to south in both oceans (Pacific and Atlantic). These records were matched into the following marine biogeographic regions (Spalding et al. 2007): Warm Temperate South-eastern Pacific (WTSP), Magellanic, Warm Temperate South-western Atlantic (WTSA) and Southern Ocean (SO). Dimensions of the polyplacophorans refer to their maximum longitude and maximum width. For gastropods, the maximum height is from the ventral umbo of the shell, and the maximum width is perpendicular to the height. Finally, for bivalves, the maximum height is from the umbo on the ventral margin, and the width is between the upper and lower margins.

In addition, larval development was investigated in order to evaluate reproductive strategy related to the dispersion mode of each species. The source of this information was: McLean (1984), Hain and Arnaud (1992), Ponder and Worsfold (1994), Osorio (2002), Pastorino (2002), Pastorino and Penchaszadeh (2002), Linse and Page (2003), Zelaya (2004), Schwabe (2009), Zelaya (2009a, 2009b), González-Wevar et al. (2011), Torroglosa and Gimenez (2012), and Liuzzi and Zelaya (2013).

### Statistical analysis

In order to detect whether our sampling effort was able to estimate the total species of molluscs, the linear dependence model was used. This was designed to estimate species richness, depending on the number of samples (Soberon and Llorente 1993). All samples were randomised so as not to affect the shape of the curve (Colwell and Coddington 1994, Moreno and Halffter 2000). The estimation method Simplex and Quasi-Newton of the statistical package STATISTICA 7 was used to estimate the coefficients of the nonlinear regression model.

Possible changes in the assemblage of molluscs throughout the year were determined using a nested design that considered each sampling site and season as sources of variation. For this, a PERMANOVA analysis was performed using species richness (Anderson 2005). Previously, the distance from Bray-Curtis similarity between pairs of observations was calculated, and 9999 permutations were used without data constraints (Anderson 2001). This analysis was developed in the FORTRAN package (Anderson 2005).

Furthermore, we defined species represented by a single individual as “singletons” and species represented by only two individuals as “doubletons” (Colwell and Coddington 1994) as a measure of species rarity.

### Analysis of biogeographic aspects

Biogeographic distribution was delimited for the species as “Widespread”, “Magellanic-Pacific southeast temperate”, “Magellanic”, “Magellanic-Atlantic southwest temperate”, and “Magellanic-Southern Ocean”, following the classification of provinces and biogeographic ecoregions proposed by Spalding et al. (2007) and taking into account previous research (Stuardo 1964, Brattström and Johanssen 1983, Lancelotti and Vásquez 2000, Camus 2001) of the Chilean Coast. In order to estimate the biogeographic affinities of the molluscs recorded in this study, a literature revision was carried out from the different provinces and regions of the South American and Antarctic coasts. A comprehensive review of the bathymetry of each species was performed. All species inhabiting depths less than 30 m were included and considered as “shallow-water species”. For the different provinces or regions of the Pacific Coast, the number of species was obtained from the revisions of Valdovinos (1999) and Ramirez et al. (2003). For the Atlantic Coast, the checklists of Scarabino (2003a, 2003b, 2004) and Carcelles (1950) were used. For the province of the Scotia Sea and continental Antarctic, the work of Griffiths et al. (2003) and a personal data compilation were used. Degrees of faunistic affinity between the studied areas were evaluated using the Simpson similarity coefficient (Cheetham and Hazel 1969), and similarities were calculated as quotient between shared species and local richness (SL; see Zelaya 2005).

## Results

From a total of 852 mollusc specimens sampled, 42 species were identified, corresponding to 9 orders, 23 families, and 31 genera. Three identities (morphotypes) were identified only at a genus level (Table 1). In terms of richness by class, Gastropoda was represented by 24 species, and Polyplacophora and Bivalvia were each represented by 9 species (Table 1). Of the total species, 38.1% were rare, with 28.6% singletons and 9.5% doubletons (Table 1). Comparing the three classes, Gastropoda had most of the rare species at 45.8% (singletons plus doubletons).

**Table 1.** Systematics list of all species of molluscs collected in quadrats and outside of them, indicating the presence (+) in the beds of Punta Santa Ana (SA) and Punta Santa and María (SM), their development mode, and rarity.

Taxon	Species	SA	SM	Development	Rarity
POLYPLACOPHORA					
Order Chitonida					
Ischnochitonidae	*Ischnochiton stramineus*	+	+	Direct	
	*Ischnochiton pusio*		+	Unknown	
Callochitonidae	*Callochiton puniceus*		+	Unknown	
Chitonidae	*Tonicia lebruni*	+	+	Direct	
	*Tonicia chilensis*		+	Unknown	
	*Tonicia atrata*		+	Unknown	
	*Chiton bowenii*	+	+	Unknown	
Mopaliidae	*Plaxiphora aurata*		+	Unknown	Singleton
	*Nuttallochiton martiali*	+		Unknown	Singleton
GASTROPODA					
Order Patellogastropoda					
Nacellidae	*Nacella deaurata*		+	Indirect	
	*Nacella flammea*	+	+	Indirect	
	*Nacella mytilina*		+	Indirect	Singleton
	*Nacella* sp		+	Indirect	
Lepetidae	*Iothia emarginuloides*		+	Unknown	
Order Vetigastropoda					
Fissurellidae	*Fissurella picta*		+	Indirect	
	*Fissurella oriens*	+	+	Indirect	
Trochidae	*Margarella violacea*	+	+	Direct	
	*Margarella expansa*	+		Direct	Singleton
Calliostomatidae	*Calliostoma nudum*		+	Unknown	Singleton
	*Calliostoma modestulum*	+		Unknown	Doubleton
	*Photinastoma taeniatum*		+	Unknown	Singleton
Order Littorinimorpha					
Calyptraeidae	*Trochita pileus*		+	Mixed	
Ranellidae	*Fusitriton magellanicus*	+		Mixed	
Eatoniellidae	*Eatoniella nigra*		+	Indirect	Singleton
Order Ptenoglosa					
Newtoniellidae	*Eumetula pulla*	+	+	Unknown	Doubleton
Order Neogastropoda					
Buccinidae	*Savatieria meridionale*		+	Unknown	Singleton
	*Pareuthria cerealis*		+	Unknown	
	*Pareuthria plumbea*	+		Direct	
	*Pareuthria paessleri*		+	Unknown	Singleton
	*Pareuthria janseni*	+		Unknown	Singleton
Muricidae	*Trophon geversianus*		+	Direct	
	*Fuegotrophon pallidus*	+	+	Direct	
	*Xymenopsis muriciformis*	+	+	Direct	
Order Heterobranchia					
Acteonidae	*Acteon biplicatus*		+	Unknown	Doubleton
BIVALVIA					
Order Pteriomorphia					
Mytilidae	*Aulacomya atra*	+	+	Indirect	
	*Mytilus edulis platensis*		+	Indirect	Singleton
Astartidae	*Astarte longirostris*		+	Indirect	
Limidae	*Limea pygmaea*		+	Direct	
Pectinidae	*Zygochlamys patagonica*		+	Indirect	Doubleton
	*Austrochlamys natans*	+		Indirect	Singleton
Philobryidae	*Philobrya* sp		+	Direct	Doubleton
Order Heterodonta					
Hiatellidae	*Hiatella* sp	+	+	Indirect	Singleton
Carditidae	*Carditella naviformis*		+	Unknown	
Veneridae	*Tawera elliptica*		+	Indirect	
Gaimardiidae	*Gaimardia trapesina*		+	Direct	

PERMANOVA analysis showed no significant differences (*F* = 0.9084; *p* = 0.6835) in the seasonal species composition of the two sites (Table 2). However, it showed significant differences (*F* = 171.972; *p* = 0.0001) in species composition between the two study sites.

**Table 2.** Analysis of permutations (PERMANOVA) of mollusc assemblages inhabiting beds of *Gigartina skottsbergii*. The sampling design was nested, considering season and sites. Data were transformed to presence/absence without permutation restrictions, based in Bray–Curtis dissimilarity analysis. The number of permutations was 9999.

Source	Df	Richness		
		Ms	*F*	*p*
Site	1	62049.77	171.972	0.0001
Site (season)	6	3277.73	0.9084	0.6835
Residual	112	3608.12		
Total	119			

**Figure 2.**
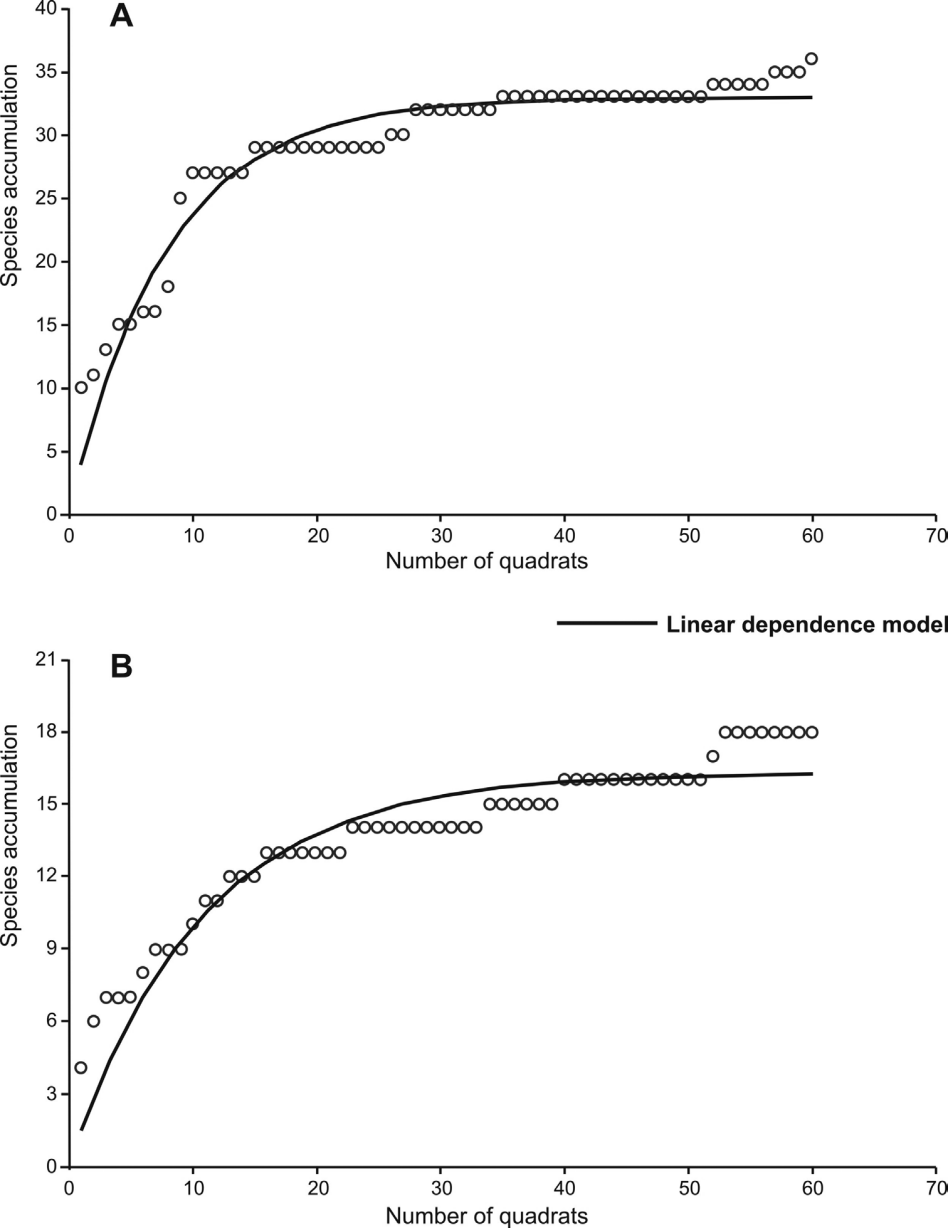
Linear dependence model to estimate the species richness associated with sampling effort in sampling sites. **A** Punta Santa Maria and **B** Punta Santa Ana.

The species richness associated with sampling effort was determined by the linear dependence model. For Punta Santa Maria, prediction constants were *a* = 0.126 and *b* = 4.179; therefore, the expected maximum richness (*a* / *b*) was 33 species with an *R^2^* = 0.96 and slope = 0.002. This value is lower than that observed in the field (*S* = 36) (Fig. 2A). Finally, for Punta Santa Ana, prediction constants were *a* = 1.522 and *b* = 0.093; therefore, the expected maximum richness (*a* / *b*) was 16 species with an *R^2^* = 0.93 and slope = 0.005. This value is lower than that observed in the field (*S* = 18) (Fig. 2B). Therefore, in this study, the richness obtained from the model of linear dependence for both sites was lower than that observed in the field.

## Systematics and distribution

### Ischnochiton stramineus

Taxon classificationAnimaliaChitonidaIschnochitonidae

(Sowerby I, 1832)

Fig. 3A

#### Material examined.

41 spm (5 × 2 – 10 × 5 mm).

#### Synonymy.

See Kaas and Van Belle (1990).

#### Remarks.

This species is capable of incubating its eggs in the pallial cavity until they are metamorphosed juveniles (Schwabe 2009). In the Strait of Magellan, Sirenko (2006) observed that the incubation period is during the month of May.

#### Distribution.

WTSP: Perú (Kaas and Van Belle 1990), Juan Fernández Islands (Plate 1899), Antofagasta (Plate 1899), Coliumo Bay and Mocha Island (Aldea and Valdovinos 2005). Magellanic: Chiloé Archipelago (Broderip and Sowerby 1832, Tryon and Pilsbry 1892), Gulf of Ancud (Leloup 1956), Punta Gaviota (Dell 1971), Estero Elefantes (Reid and Osorio 2000), and Puerto Edén (Dell 1971); Strait of Magellan (Plate 1899, Dell 1964, Sirenko 2006): Punta Santa María (Leloup 1956; this record) and Carlos III Island (Aldea et al. 2011a); Cockburn Channel (Plate 1899), London Island (Pelseneer 1903), Beagle Channel (Plate 1899), Hermite Islands (Dell 1971), Seno Grandi (Dell 1971), Malvinas/Falkland Islands (Kass and Van Belle 1990, Sirenko 2006), Tierra del Fuego (Sirenko 2006), and Staten Island (Sirenko 2006). SO: South Georgia Island (Kass and Van Belle 1990).

**Figure 3.**
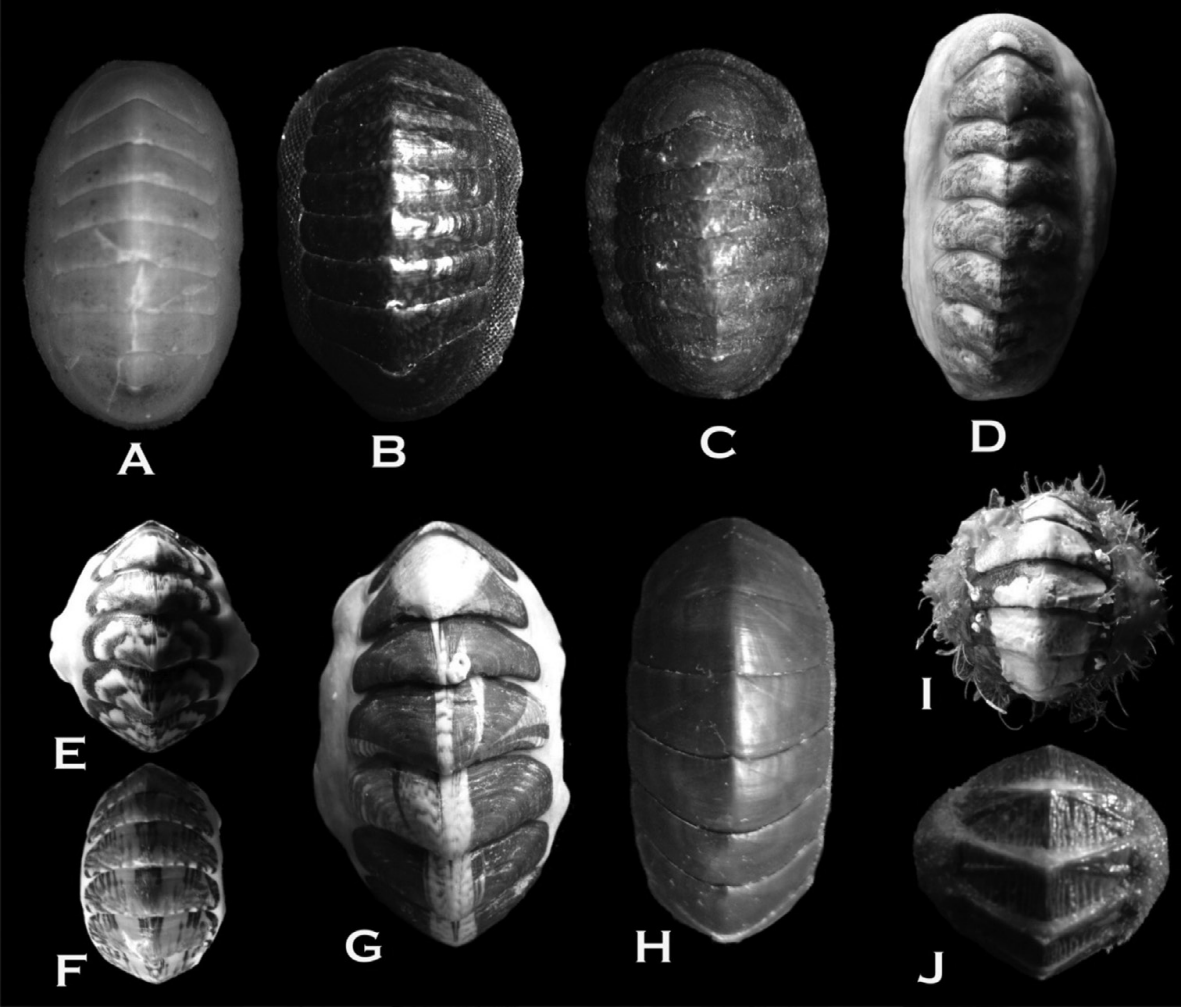
**A** *Ischnochiton stramineus* (10 × 5 mm **B** *Ischnochiton pusio* (11 × 6 mm) **C** *Callochiton puniceus* (11 × 6 mm) **D** *Tonicia lebruni* (25 × 13 mm) **E–F** *Tonicia chilensis* (20 × 10 mm and 22 × 11 mm) **G** *Tonicia atrata* (50 × 26 mm) **H** *Chiton bowenii* (26 × 13 mm) **I** *Plaxiphora aurata* (30 × 20 mm) **J** *Nuttallochiton martiali* (20 × 10 mm).

### Ischnochiton pusio

Taxon classificationAnimaliaChitonidaIschnochitonidae

(Sowerby I, 1832)

Fig. 3B

#### Material examined.

3 spm (11 × 6 – 16 × 9 mm).

#### Synonymy.

See Kaas and Van Belle (1994).

#### Remarks.

Regarding its habits, Reid and Osorio (2000) commented that *Ischnochiton pusio* inhabits the lower part of the rocks and other hard substrates in the intertidal zone up to 90 m, with a depth preference between 5 and 30 m in the fjord zones. Consequently, Schwabe et al. (2006) mentioned that this species is less tolerant of the fluctuations in salinity, and for that reason, inhabits below the halocline.

#### Distribution.

WTSP: Perú (Kass and Van Belle 1994), Juan Fernández Islands (Kass and Van Belle 1994), Antofagasta (Plate 1899), Valparaíso (Broderip and Sowerby 1832) and Talcahuano (Plate 1899). Magellanic: Puerto Montt (Plate 1899), Gulf of Ancud (Leloup 1956), Reloncaví Sound (Leloup 1956), Comau Fjord (Schwabe et al. 2006), Estero Elefantes (Reid and Osorio 2000), Puerto Edén (Dell 1971), and Smyth Channel (Castellanos 1956); Strait of Magellan (Castellanos 1956): Punta Santa María (this record) and Carlos III Island (Aldea et al. 2011a).

### Callochiton puniceus

Taxon classificationAnimaliaChitonidaCallochitonidae

(Gould, 1846)

Fig. 3C

#### Material examined.

76 spm (2 × 2.5 – 11 × 6 mm).

#### Synonymy.

See Kaas and Van Belle (1985).

#### Remarks.

Morphologically, this species is similar to *Stenosemus exaratus* (G.O. Sars, 1878) but differs by presenting a wider perinotum, black pigmented aesthetes, and different elements of the dorsal perinotum (Schwabe 2009). In relation to its colour, Sirenko (2006) mentioned that it can vary from white to red.

#### Distribution.

Magellanic: Puerto Montt (Dell 1971), Gulf of Corcovado (Cárdenas et al. 2008), and Puerto Edén (Dell 1971); Strait of Magellan (Dell 1964, Sirenko 2006): eastern micro-basin of the Strait of Magellan (Ríos et al. 2003), Punta Santa María (this record), Inútil Bay (USNM 2010), Cape Froward (USNM 2010), and Carlos III Island (Aldea et al. 2011a); Puerto Williams (Dell 1971), Róbalo Bay (Dell 1971), Orange Bay (Rochebrune and Mabille 1889), Hermite Islands (Dell 1971), Seno Grandi (Dell 1971), and Cape Horn (Kaas and Van Belle 1985, USNM 2010); Chubut (Bigatti 2010), Malvinas/Falkland Islands (Dell 1964, Sirenko 2006), and Staten Island (Sirenko 2006). SO: Queen Maud Land (Smirnov et al. 2000), Enderby Land (Smirnov et al. 2000), and Amery Ice-Shelf (Constable et al. 2007).

### Tonicia lebruni

Taxon classificationAnimaliaChitonidaChitonidae

(Rochebrune, 1884)

Fig. 3D

#### Material examined.

117 spm (12 × 6 – 32 × 16 mm).

#### Synonymy.

See Kaas et al. (2006).

#### Remarks.

Frequently, it was considered a synonym for the species *Tonicia calbucensis* Plate, 1898, until *Tonicia calbucensis* was re-established as a valid species, based on six different characteristics between both species (Schwabe et al. 2006, p. 15). Sirenko (2006) stated that this species incubates its eggs in the pallial cavity until juvenile, and consequently in this study, a sample from the winter season was found with juveniles in the pallial cavity.

#### Distribution.

Magellanic: Gulf of Ancud (Leloup 1956) and Puerto Edén (Dell 1971); Strait of Magellan (Rochebrune and Mabille 1889, Tryon and Pilsbry 1892, Sirenko 2006): eastern micro-basin of the Strait of Magellan (Ríos et al. 2003), Punta Arenas (Rochebrune and Mabille 1889, Tryon and Pilsbry 1892), Río de los Ciervos (Leloup 1956), Punta Santa Ana (this record), Punta Santa María (Leloup 1956; this record), Inútil Bay (Thiele 1908), and Carlos III Island (Aldea et al. 2011a); Beagle Channel (Thiele 1908), Ushuaia (Thiele 1908), Puerto Williams, Róbalo Bay, Hermite Islands, Bertrand Island and Seno Grandi (Dell 1971), Basket Island (Thiele 1908), Orange Bay (Rochebrune and Mabille 1889, Tryon and Pilsbry 1892), and Puerto Toro (Thiele 1908); from the Chubut Province southward (Sirenko 2006), Malvinas/Falkland Islands (Sirenko 2006), Tierra del Fuego (Sirenko 2006), and Staten Island (USNM 2010).

### Tonicia chilensis

Taxon classificationAnimaliaChitonidaChitonidae

(Frembly, 1827)

Fig. 3E–F

#### Material examined.

40 spm (9 × 5 – 26 × 13 mm).

#### Synonymy.

See Kaas et al. (2006).

#### Remarks.

Sirenko (2006) did not include this species within the list of Magellanic Polyplacophora. However, Schwabe et al. (2006) named it as *Tonicia chilensis* for all individuals that presented the following characteristics: well-marked micro-granulations in all of the valves, granules that are marked towards the margins of the valves, characteristics that other species of the genus did not present, except *Tonicia lebruni*. Our specimens did present granules but disposed in an irregular form, and *Tonicia calbucencis* sometimes can present granules along the entirety of the margins (Schwabe et al. 2006).

#### Distribution.

WTSP: Perú (Leloup 1956), Valparaíso (Leloup 1956), Montemar (Leloup 1956), Punta Pingueral and Cape Tirúa (Aldea and Valdovinos 2005), Gulf of Arauco (Leloup 1956) and Valdivia (Zagal and Hermosilla 2001). Magellanic: Comau Fjord (Schwabe et al. 2006), Punta Pulga (Dell 1971), Gulf of Ancud (Leloup 1956), and Estero Elefantes (Reid and Osorio 2000); Strait of Magellan: eastern micro-basin of the Strait of Magellan (Ríos et al. 2003) and Punta Santa María (this record); Róbalo Bay (Ojeda et al. 2010).

### Tonicia atrata

Taxon classificationAnimaliaChitonidaChitonidae

(Sowerby II, 1840)

Fig. 3G

#### Material examined.

7 spm (17 × 8 – 50 × 26 mm).

#### Synonymy.

See Kaas et al. (2006).

#### Remarks.

Sirenko (2006) mentioned that the coloring of the valves of this species is variable. It is known that this species houses the protozoa parasite *Chitonicum simplex* Plate 1898 (Schwabe et. al 2006).

#### Distribution.

WTSP: between Punta Pingueral and Cape Tirúa (Aldea and Valdovinos 2005) and Valdivia (Zagal and Hermosilla 2001). Magellanic: Comau Fjord (Schwabe et al. 2006), Punta Pulga (Dell 1971), Gulf of Ancud (Leloup 1956), Chonos Archipelago (Leloup 1956), Estero Elefantes (Reid and Osorio 2000), and Puerto Edén (Dell 1971); Strait of Magellan (Sirenko 2006): eastern micro-basin of the Strait of Magellan (Ríos et al. 2003), Laredo Bay (Mutschke et al. 1998), Punta Arenas (Thiele 1908), Río de los Ciervos (Leloup 1956), Porvenir (Thiele 1908), Punta Santa María (this study), Punta Santa Ana (Ríos et al. 2007), and Carlos III Island (Aldea et al. 2011a); Smyth Channel (Thiele 1908), London Island (Pelseneer 1903), Beagle Channel (Pelseneer 1903), Ushuaia (Thiele 1908), Puerto Williams (Dell 1971), Róbalo Bay (Dell 1971, Ojeda et al. 2010), Bertrand Island (Dell 1971), and Seno Grandi (Dell 1971); Malvinas/Falkland Islands (Sowerby 1840, Tryon and Pilsbry 1892, Melvill and Standen 1914, Sirenko 2006).

### Chiton bowenii

Taxon classificationAnimaliaChitonidaChitonidae

(King & Broderip, 1831)

Fig. 3H

#### Material examined.

15 spm (13 × 7 – 29 × 15 mm).

#### Synonymy.

See Kaas et al. (2006).

#### Remarks.

Sirenko (2006) commented that *Chiton bowenii* is a rare species. However, in this study, it was present in two sampling sites.

#### Distribution.

Magellanic: Strait of Magellan (King and Broderip 1832, Sirenko 2006): Laredo Bay (Sirenko 2006), Punta Santa Ana (Sirenko 2006; this record), Punta Santa María (this record), and Carlos III Island (Aldea et al. 2011a); Orange Bay (Rochebrune and Mabille 1889).

### Plaxiphora aurata

Taxon classificationAnimaliaChitonidaMopaliidae

(Spalowsky, 1795)

Fig. 3I

#### Material examined.

1 spm (30 × 20 mm).

#### Synonymy.

See Kaas and Van Belle (1994).

#### Remarks.

Reid and Osorio (2000) mentioned that this species together with the tiny species *Leptochiton medinae* (Plate, 1899) are the only chitons capable of inhabiting environments with low salinity. Morphologically, this species is distinguished by presenting variable coloring in the valves and in the tegument sculpture (Sirenko 2006).

#### Distribution.

WTSP: Valparaíso (King and Broderip 1832), between Punta Pingueral and Cape Tirúa (Aldea and Valdovinos 2005). Magellanic: Gulf of Ancud (Leloup 1956), Estero Elefantes (Reid and Osorio 2000), Puerto Edén (Dell 1971), Paso de Indio (Dell 1971), and Piazzi Island (Dell 1971); Strait of Magellan (Dell 1964): Buque Quemado (Aldea and Rosenfeld 2011), eastern micro-basin of the Strait of Magellan (Ríos et al. 2003), Laredo Bay (Mutschke et al. 1998), Punta Arenas (Rochebrune and Mabille 1889), Río de los Ciervos (Leloup 1956), Punta Santa Ana (Ríos et al. 2007), Punta Santa María (Leloup 1956; this record), and Carlos III Island (Aldea et al. 2011a); Cockburn Channel (Dell 1964), Puerto Williams (Dell 1971), Róbalo Bay (Dell 1971, Ojeda et al. 2010), Hermite Islands (Dell 1971), Bertrand Island (Dell 1971), and Orange Bay (Rochebrune and Mabille 1889); Malvinas/Falkland Islands (Dell 1964, Sirenko 2006), San Sebastián Bay (USNM 2010), and Staten Island (Sirenko 2006, USNM 2010). SO: Also reported for Antarctica (Götting 1989).

### Nuttallochiton martiali

Taxon classificationAnimaliaChitonidaMopaliidae

(Rochebrune in Rochebrune & Mabille, 1889)

Fig. 3J

#### Material examined.

1 spm (20 × 10 mm).

#### Synonymy.

See Kaas and Van Belle (1987).

#### Remarks.

This species presents a morphological similarity to *Plaxiphora aurata* but presents longitudinal elevations in the pleural areas, while *Plaxiphora aurata* does not possess this sculpture (Schwabe 2009). According to Sirenko (2006), it is a rare species.

#### Distribution.

Magellanic: Gulf of Corcovado (Cárdenas et al. 2008) and Comau Fjord (Schwabe et al. 2006); Strait of Magellan (Leloup 1956): Punta Santa Ana (this record) and Carlos III Island (Aldea et al. 2011a); Róbalo Bay (Dell 1971); Malvinas/Falkland Islands (Dell 1964, Sirenko 2006) and Staten Island (Sirenko 2006).

### Nacella deaurata

Taxon classificationAnimaliaNot assignedNacellidae

(Gmelin, 1791)

Fig. 4A–B

#### Material examined.

66 spm (17 × 12 × 8 – 21 × 17 × 11 mm).

#### Synonymy.

See Valdovinos and Rüth (2005).

#### Remarks.

According to the classification done by Valdovinos and Rüth (2005), the shell morphology of *Nacella deaurata* is similar to the species *Nacella delicatissima*. Later, Arazamendi et al. (2009), based on molecular techniques, concluded that the specimens of *Nacella delicatissima* are combined with the specimens of *Nacella magellanica* and *Nacella deaurata*, suggesting that *Nacella delicatissima* is a morphotype of both species.

#### Distribution.

Magellanic: Apiao Archipelago (Valdovinos and Rüth 2005), Estero Elefantes (Reid and Osorio 2000), and Summer Island (Valdovinos and Rüth 2005); Strait of Magellan (Tryon and Pilsbry 1891, Powell 1951): Buque Quemado (Aldea and Rosenfeld 2011), eastern micro-basin of the Strait of Magellan (Ríos et al. 2003), Laredo Bay (Mutschke et al. 1998, Valdovinos and Rüth 2005, Gónzalez-Wevar et al. 2010), Punta Santa María (Valdovinos and Rüth 2005; this record), Punta Santa Ana (Valdovinos and Rüth 2005), Águila Bay (Gónzalez-Wevar et al. 2010), Caleta Carden, Leñadura Beach, Punta Arenas and Punta Chilota (Valdovinos and Rüth 2005), Dawson Island (Ramírez 1996, USNM 2010), and Carlos III Island (Aldea et al. 2011a); Beagle Channel and Ushuaia (Aranzamendi et al. 2009), Puerto Williams (Valdovinos and Rüth 2005), Róbalo Bay (Ojeda et al. 2010), Orange Bay and Hoste Island (Valdovinos and Rüth 2005), Cape Horn (Rochebrune and Mabille 1889), Diego Ramírez Islands (Valdovinos and Rüth 2005) and Malvinas/Falkland Islands (González-Wevar et al. 2011). WTSA: from 38°S toward south (Morris and Rosenberg 2005). SO: Kerguelen Islands (Lamy 1905, Carcelles 1950).

**Figure 4.**
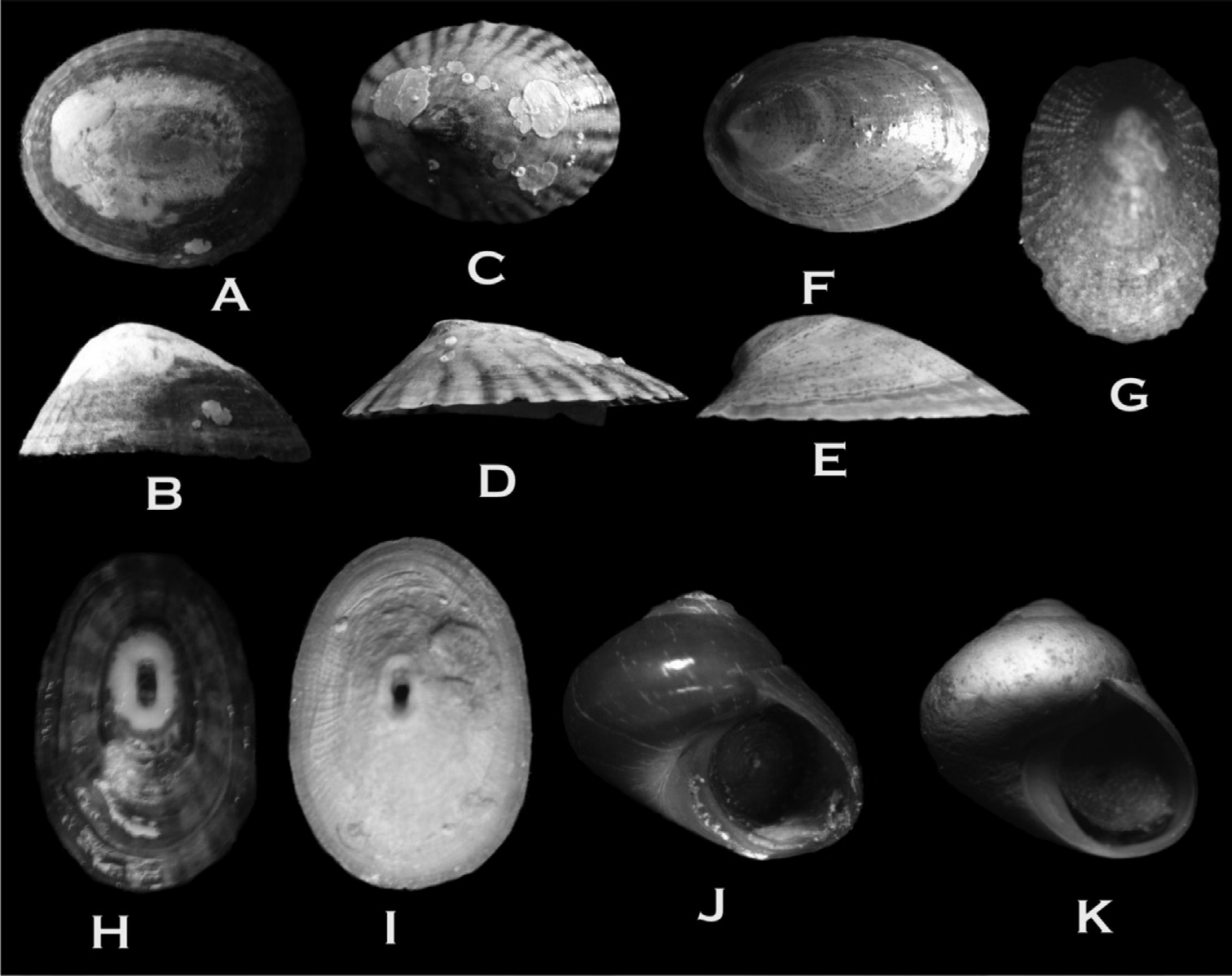
**A–B** *Nacella deaurata* (17 × 12 × 8 mm) **C–D** *Nacella flammea* (45 × 33 × 10 mm) **E–F** *Nacella mytilina* (26 × 18 × 10 mm) **G** *Iothia emarginuloides* (4 × 2.3 × 1.5 mm) **H** *Fissurella picta picta* (19 × 16 × 10) **I** *Fissurella oriens* (43 × 32 × 16 mm) **J** *Margarella violacea* (9 × 9 mm) **K** *Margarella expansa* (7.5 × 8 mm).

### Nacella flammea

Taxon classificationAnimaliaNot assignedNacellidae

(Gmelin, 1791)

Fig. 4C–D

#### Material examined.

19 spm (9 × 7 × 3 – 45 × 33 × 10 mm).

#### Synonymy.

See Valdovinos and Rüth (2005).

#### Remarks.

*Nacella flammea* and *Nacella mytilina* inhabit subtidal environments. *Nacella flammea* presents a shell morphology different from the rest of the species of the genus (Valdovinos and Rüth 2005). This species mainly inhabits rocky bottoms, feeding on benthic microalgae (Gonzalez-Wevar et al. 2011).

#### Distribution.

Magellanic: Aysén (Valdovinos and Rüth 2005) and Guarello Island (Valdovinos and Rüth 2005); Strait of Magellan: Laredo Bay (Mutschke et al. 1998), Punta Santa Ana (Gónzalez-Wevar et al. 2010), Punta Santa María (this record), Carlos III Island (Aldea et al. 2011a), and Almirantazgo Sound (Valdovinos and Rüth 2005); Róbalo Bay (Ojeda et al. 2010).

### Nacella mytilina

Taxon classificationAnimaliaNot assignedNacellidae

(Helbling, 1779)

Fig. 4E–F

#### Material examined.

1 spm (26 × 18 × 10 mm).

#### Synonymy.

See Valdovinos and Rüth (2005).

#### Remarks.

This species was recorded as a junior synonym of *Nacella kerguelensis* by Cantera and Arnaud (1985). Nevertheless, Valdovinos and Rüth (2005) commented that morphologically *Nacella mytilina* is clearly different from the rest of the species. The molecular study carried out by Gonzalez-Wevar et al. (2010) backed the establishment of *Nacella mytilina* and *Nacella kerguelensis* as different species. *Nacella mytilina* is a common component of the epibiontic community associated with *Macrocystis pyrifera* kelp forests of the Magellan Region (Reid and Osorio 2000). In this study, it was found inhabiting the fronds of *Gigartina skottsbergii*.

#### Distribution.

Magellanic: Estero Elefantes (Reid and Osorio 2000), Carlos Island in Puerto Edén (Dell 1971), and Guarello Island (Valdovinos and Rüth 2005); Strait of Magellan (Tryon and Pilsbry 1891): Punta Arenas (Valdovinos and Rüth 2005), Punta Chilota (Valdovinos and Rüth 2005), Punta Santa Ana (Gónzalez-Wevar et al. 2010), Punta Santa María (this record), Dawson Island (Valdovinos and Rüth 2005, USNM 2010), Magdalena Island (Pelseneer 1903), Carlos III Island (Gónzalez-Wevar et al. 2010, Aldea et al. 2011a), Puerto Hope (Pelseneer 1903), and McClelland River in Tierra del Fuego (Smith 1905); London Island (Pelseneer 1903), Beagle Channel (Pelseneer 1903), Puerto Williams (Dell 1971), Puerto Róbalo (Dell 1971), Puerto Harberton, Bertrand Island (Dell 1971), Puerto Deseado (Aranzamendi et al. 2009), and Staten Island (Pelseneer 1903, USNM 2010). WTSA: from 39°S toward south (Carcelles 1950). SO: Kerguelen Islands (Smith 1879, Tryon and Pilsbry 1891, Thiele 1912, Troncoso et al. 2001).

### Iothia emarginuloides

Taxon classificationAnimaliaNot assignedLepetidae

(Philippi, 1868)

Fig. 4G

#### Material examined.

13 spm (3 × 2 × 1 – 4 × 2.3 × 1.5 mm).

#### Synonymy.

See Waren et al. (2011).

#### Remarks.

Waren et al. (2011) studied the species of Lepetidae and concluded that specimens of *Iothia coppingeri* and *Iothia emarginuloides* are similar. This is concurrent with Strebel (1907) that these species are synonyms, establishing *Iothia copperingeri* as a junior synonym of this species.

#### Distribution.

WTSP: Valdivia (Dell 1990). Magellanic: Gulf of Ancud (Waren et al. 2011), Chiloé Archipelago (Cárdenas et al. 2008), and Estero Elefantes (Reid and Osorio 2000); Strait of Magellan (Smith 1881, Dell 1990): Punta Arenas (Thiele 1912, Waren et al. 2011), eastern micro-basin of the Strait of Magellan (Ríos et al. 2003), Punta Santa María (this record), and Carlos III Island (Aldea et al. 2011a); Beagle Channel (Linse 1997) and Cape Horn (Rochebrune and Mabille 1889); Malvinas/Falkland Islands (Strebel 1908, Powell 1951) and Staten Island (Dell 1990). SO: South Georgia Island (Zelaya 2005), South Orkney Islands (Dell 1990), Weddell Sea (Dell 1990, Hain 1990, Gutt et al. 2003), Ross Sea (USNM 2010, OBIS 2014), South Shetland Islands (Dell 1990, Aldea and Troncoso 2008), Kerguelen Islands (Powell 1957, Cantera and Arnaud 1985), Macquaire Island (USNM 2010), Wilkes Land (USNM 2010), and Enderby Land (Powell 1958).

### Fissurella picta picta

Taxon classificationAnimaliaNot assignedFissurellidae

(Gmelin, 1791)

Fig. 4H

#### Material examined.

3 spm (19 × 16 × 10 – 38 × 25 × 14 mm).

#### Synonymy.

See McLean (1984).

#### Remarks.

Aldea and Rosenfeld (2011) commented that it is generally similar to *Fissurella radiosa radiosa* Lesson, 1831, sharing geographic distribution points. McLean (1984) mentioned the characteristics that differentiate them: *Fissurella picta* presents more wide shell margins in all of its development stages and the foramen is more centralized and presents black rays that do not occur on *Fissurella radiosa*.

#### Distribution.

WTSP: Valparaíso (Hupé 1854, Tryon and Pilsbry 1890, McLean 1984), Papudo (Ramírez 1996), Punta Pingueral and Cape Tirúa (Aldea and Valdovinos 2005), and Valdivia (Zagal and Hermosilla 2001). Magellanic: Chiloé Archipelago (McLean 1984), Estero Elefantes (Reid and Osorio 2000), and Puerto Edén (Dell 1971); Strait of Magellan (Rochebrune and Mabille 1889): Buque Quemado (Aldea and Rosenfeld 2011), Laredo Bay (Mutschke et al. 1998), Punta Arenas (Rochebrune and Mabille 1889), Punta Santa Ana (Ríos et al. 2007), Punta Santa María (this record), Inútil Bay (Ramírez 1996), and Carlos III Island (Aldea et al. 2011a); Ushuaia (Strebel 1908), Puerto Williams (Dell 1971), Róbalo Bay (Dell 1971, Ojeda et al. 2010), Orange Bay (Rochebrune and Mabille 1889), and Navidad Bay (Ramírez 1996); Malvinas/Falkand Islands (Rochebrune and Mabille 1889, Melvill and Standen 1914) in Port Stanley (Watson 1886, Powell 1951).

### Fissurella oriens

Taxon classificationAnimaliaNot assignedFissurellidae

Sowerby I, 1834

Fig. 4I

#### Material examined.

62 spm (12 × 8 × 4 – 43 × 32 × 16 mm).

#### Synonymy.

See McLean (1984).

#### Remarks.

According to McLean (1984), the most similar species is *Fissurella radiosa*, which is similar in size and presents similar colors and variations. The same author comments that the only distinguishing characteristic between the shells is the presence of primary ribs that are longer than the adjacent ribs present in *Fissurella radiosa*. These primary ribs are absent in the species *Fissurella oriens*.

#### Distribution.

WTSP: Mehuín (McLean 1984). Magellanic: Chiloé Archipelago (Dall 1909), Calbuco (Ramirez 1996), Queullín Island (Ramirez 1996), Punta Chulao (Ramirez 1996), Estero Elefantes (Reid and Osorio 2000), Puerto Edén and Wellington Island (Dell 1971), Carlos Island (Dell 1971), Levinzon Island (Dell 1971), Piazzi Island (Dell 1971), Melchior Island (Ramirez 1996), and Smyth Channel (Strebel 1907); Strait of Magellan (Húpe 1854, Tryon and Pilsbry 1890, Ramírez 1996): Laredo Bay (Mutschke et al. 1998), Punta Arenas, (Rochebrune and Mabille 1889, Strebel 1907), Punta Santa Ana (Ríos et al. 2007; this record), Porvenir (Strebel 1907), Punta Santa María (this record), Inútil Bay (Strebel 1907), Carlos III Island (Aldea et al. 2011a), Puerto Churruca (Strebel 1907), Puerto Angosto (Strebel 1907), and Borja Bay (Strebel 1907); Ushuaia (Strebel 1907), Puerto Williams (Dell 1971), Hermite Islands (Dell 1971), Seno Grandi (Dell 1971), Basket Island (Strebel 1907), Picton Island (Strebel 1907), Orange Bay (Rochebrune and Mabille 1889), and Cape Horn (Rochebrune and Mabille 1889); Santa Cruz (Osorio 1999), Malvinas/Falkland Islands (Melvill and Standen 1907, Powell 1951) in Port Stanley (Strebel 1907, Strebel 1908), Lively Island (Strebel 1907), and Port Albemarle (Strebel 1908).

### Margarella violacea

Taxon classificationAnimaliaNot assignedCalliostomatidae

(King & Broderip, 1831)

Fig. 4J

#### Material examined.

69 spm (3 × 2.5 – 9 × 9 mm).

#### Synonymy.

See Dell (1971).

#### Remarks.

Rosenfeld et al. (2011) commented that the shell of the similar species *Margarella expansa* (Sowerby I, 1838) is composed of two well-differentiated layers, with the internal layer being thicker. Also, *Margarella expansa* have four pairs of epipodial tentacles and frequently present an additional unpaired tentacle (Zelaya 2004). However, the identification between these species is quite complex due to the extreme morphological similarities (see Zelaya 2004, Rosenfeld et al. 2011). In this sense, Troncoso et al. (2001, p. 86) recorded and commented on *Margarella violacea* for the Kerguelen Islands, but in fact they photographed and mentioned *Margarella expansa* (Troncoso et al. 2001, p. 89, Fig. 4).

#### Distribution.

Magellanic: Estero Elefantes (Reid and Osorio 2000), Puerto Edén (Dell 1971), Nelson Strait (Linse 2002), Kirke Channel (Linse 2002), and Smyth Channel (Strebel 1905a); Strait of Magellan (King and Broderip 1832, Strebel 1905a): eastern micro-basin of the Strait of Magellan (Ríos et al. 2003), Punta Arenas (Strebel 1905a), Agua Fresca (Strebel 1905a), Cape Valentín (Strebel 1905a), Porvenir (Strebel 1905a), Inútil Bay (USNM 2010), Gente Grande Bay (Strebel 1905a), Punta Santa María (this record), Punta Santa Ana (Ríos et al. 2007; this record), Dawson Island (Strebel 1905a, USNM 2010), and Carlos III Island (Aldea et al. 2011a); Elizabeth Island (Strebel 1905a), Beagle Channel (Rochebrune and Mabille 1889), Ushuaia (Strebel 1905a, 1908), Navarino Island (Strebel 1905a), Puerto Williams (Dell 1971), Róbalo Bay (Dell 1971, Ojeda et al. 2010), Basket Island (Strebel 1905a), Puerto Toro (Strebel 1905a), Orange Bay (Rochebrune and Mabille 1889), Seno Grandi (Dell 1971), Goree Passage (Linse 2002), Picton Island (Strebel 1905a, Linse 2002), Lenox Island (Strebel 1905a), Bertrand Island (Dell 1971), and Cape Horn (Gould 1852, Linse 2002); Malvinas/Falkland Islands (Strebel 1905a, Powell 1951), Burdwood Bank (Melvill and Standen 1907), and Staten Island (USNM 2010).

### Margarella expansa

Taxon classificationAnimaliaNot assignedCalliostomatidae

(Sowerby I, 1838)

Fig. 4K

#### Material examined.

1 spm (7.5 × 8 mm).

#### Synonymy.

See Powell (1951).

#### Remarks.

New information about the biology and distribution of the species was presented by Rosenfeld et al. (2011). They noted that the records made by Strebel (1908) for the South Georgia and South Sandwich Islands and the Antarctic Peninsula and records made by Smith (1902) for eastern Antarctica have not been commented by any other author in later studies. Because of this, these authors consider their Antarctic distribution points as dubious records, manifesting that this species would be restricted to Subantarctic regions.

#### Distribution.

Magellanic: Strait of Magellan: Buque Quemado (Aldea and Rosenfeld 2011), Puerto del Hambre (Sowerby 1838), Capitán Aracena Island (Rosenfeld et al. 2011), and Carlos III Island (Aldea et al. 2011a); Ushuaia (Zelaya 2004), Róbalo Bay (Rosenfeld et al. 2011), and Orange Bay (Lamy 1905); Malvinas/Falkland Islands (Melvill and Standen 1898, Strebel 1905a, Castellanos and Landoni 1989), and Burdwood Bank (Melvill and Standen 1907). WTSA: Río de la Plata basin (USNM 2010). SO: Marion and Prince Edward Islands (Watson 1886, Branch et al. 1991), Kerguelen Islands (Smith 1879, Watson 1886, Martens and Thiele 1904, Strebel 1905a, Thiele 1912, Lamy 1915, Powell 1957, Cantera and Arnaud 1985), and Crozet Island (Cantera and Arnaud 1985); probably in South Georgia Island (Strebel 1908), Antarctic Peninsula (Strebel 1908), and Cape Adare (Smith 1902).

### Calliostoma nudum

Taxon classificationAnimaliaNot assignedCalliostomatidae

(Philippi, 1845)

Fig. 5A

#### Material examined.

1 spm (13 × 12 mm).

#### Synonymy.

See Morris and Rosenberg (2005).

#### Remarks.

Castellanos and Landoni (1989) commented that this species is a complex variable in which the species *Calliostoma kophameli* Strebel, 1905, *Calliostoma venustulum* (Strebel, 1908), and *Calliostoma falklandicum* (Strebel, 1908) appear to be simply different morphotypes of the species *Calliostoma nudum*. Accordingly, a morphological study is required that details the various examples of the species.

#### Distribution.

Magellanic: Strait of Magellan (Castellanos and Fernández 1976): eastern micro-basin of the Strait of Magellan (Ríos et al. 2003) and Punta Santa María (this record); Beagle Channel (Osorio 1999) and Cape Horn (Osorio 1999, Linse 2002); Malvinas/Falkland Islands (Rochebrune and Mabille 1889, Strebel 1905a, 1908), Port Albemarle (Strebel 1908), Le Maire Strait (Strebel 1905a), and Staten Island (Castellanos and Landoni 1989). WTSA: At 38°S (Castellanos and Fernández 1976).

**Figure 5.**
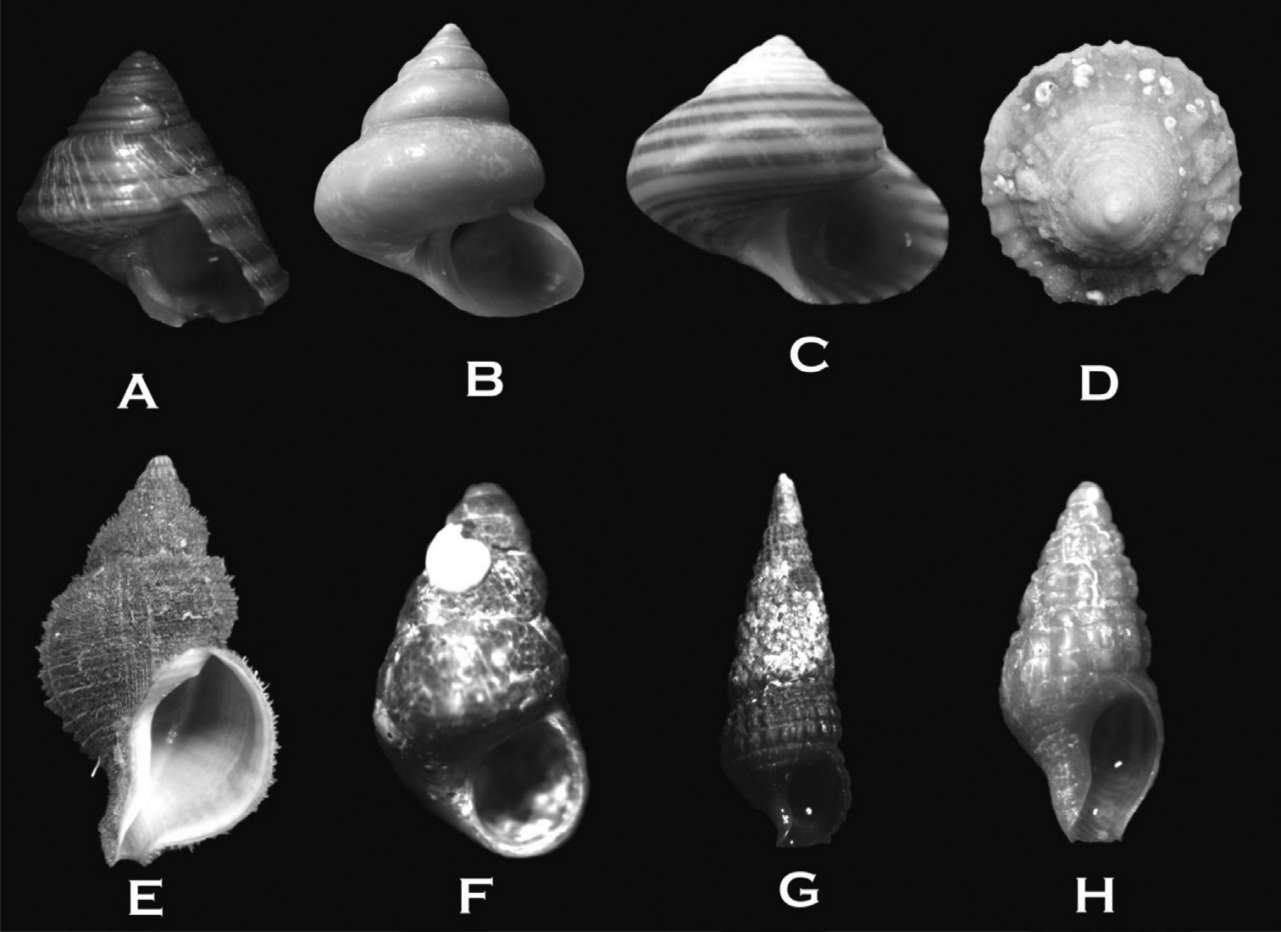
**A** *Calliostoma nudum* (13 × 12 mm) **B** *Calliostoma modestulum* (15 × 13 mm) **C** *Photinastoma taeniatum* (10 × 12 mm) **D** *Trochita pileus* (22 × 10 mm) **E** *Fusitriton magellanicus* (84 × 40 mm) **F** *Eatoniella nigra* (2 × 1 mm) **G** *Eumetula pulla* (7 × 3 mm) **H** *Savatieria meridionalis* (5 × 2 mm).

### Calliostoma modestulum

Taxon classificationAnimaliaNot assignedCalliostomatidae

(Strebel, 1908)

Fig. 5B

#### Material examined.

2 spm (13 × 11 – 15 × 13 mm).

#### Synonymy.

*Calliostoma modestulum* (Strebel, 1908): 70, pl. I, figs. 13a–b; Melvill and Standen 1912: 347. Carcelles and Williamson 1951: 263, Castellanos and Landoni 1989: 8, pl. I, fig. 8.

*Calliostoma modestula*, Castellanos and Fernández 1976: 141, pl. II, figs. 8–9.

#### Remarks.

From a morphological point of view, Melvill and Standen (1912) commented that this species presents similarities to *Photinula crawshayi* (Smith, 1905), although it has more globular whorls. The maximum depth at which it has been recorded is 869 m. However, in this study, a shallower depth was recorded, with specimens found at 10 m in beds of *Gigartina skottsbergii*.

#### Distribution.

Magellanic: Strait of Magellan: Punta Santa Ana (this record) and western entrance of the Strait of Magellan (USNM 2010); Cockburn Channel (Powell 1951); from Chubut (Castellanos and Fernández 1976), Malvinas/Falkland Islands (Strebel 1908, Powell 1951, Castellanos and Fernández 1976), and Burdwood Bank (Melvill and Standen 1912).

### Photinastoma taeniatum

Taxon classificationAnimaliaNot assignedCalliostomatidae

(Sowerby I, 1825)

Fig. 5C

#### Material examined.

1 spm (10 × 12 mm).

#### Synonymy.

See Powell (1951).

#### Remarks.

Powell (1951) stated that the subspecies *Photinastoma taeniatum nivea* Cooper & Preston, 1910 presented uncommon characteristics compared to the typical form of the species, not presenting the same color pattern and a higher spire with more globular whorls, but both forms have three spiral whorls in the first whorl of the protoconch. Similarly, Castellanos and Landoni (1989) mentioned that these characteristics had been used by Powell (1951) to identify difference on generic level between those species of *Photinastoma* and *Calliostoma*, which were similar. Given this, they estimated that the species should be included within the genus *Calliostoma*. However, according to Rosenberg (2012), this species should be included under the genus *Photinastoma*.

#### Distribution.

Magellanic: Strait of Magellan (Castellanos and Landoni 1989, Ríos et al. 2003): Punta Arenas (Rochebrune and Mabille 1889), Punta Santa María (this record), and western entrance of the Strait of Magellan (Osorio 1999); Santa Cruz River (Powell 1951) and Malvinas/Falkland Islands (Strebel 1908) in Port Stanley (Strebel 1908, Powell 1951). SO: South Georgia Island (Davolos and Moolenbeeck 2005).

### Trochita pileus

Taxon classificationAnimaliaLittorinimorphaCalyptraeidae

(Lamark, 1822)

Fig. 5D

#### Material examined.

94 spm (2 × 1 – 22 × 10 mm).

#### Synonymy.

See Linse (2002).

#### Remarks.

This species has a very similar external morphology to *Trochita pileolus* (d’Orbigny, 1984). Aldea and Rosenfeld (2011) explained that the most conspicuous external difference is that *Trochita pileus* has a smoother protoconch while *Trochita pileolus* has a wrinkled protoconch. Reid and Osorio (2000) denied the presence of the species *Trochita trochiformis* in Tierra del Fuego and the Strait of Magellan, previously reported by Carcelles and Williamson (1951), claiming that this record was probably referring to the species *Trochita pileus*.

#### Distribution.

WTSP: Santa María Island (Aldea and Valdovinos 2005), Lebu (Aldea and Valdovinos 2005), and Mocha Island (Aldea and Valdovinos 2005). Magellanic: Strait of Magellan (Aldea and Rosenfeld 2011): Laredo Bay (Mutschke et al. 1998, Linse 2002), eastern micro-basin of the Strait of Magellan (Osorio 1999, Linse 2002, Ríos et al. 2003), Cape Froward (Osorio 1999), and Voces Bay (Linse 2002); Punta Rico (Linse 2002), Picton Island (Linse 2002), Gardiner Island (Linse 2002), Brecknock Channel (Linse 2002), Beagle Channel (Osorio 1999), Goree Passage (Linse 2002), and Staten Island (USNM 2010). WTSA: in Buenos Aires Province (Strebel 1908).

### Fusitriton magellanicus

Taxon classificationAnimaliaLittorinimorphaRanellidae

(Röding, 1798)

Fig. 5E

#### Material examined.

5 spm (82 × 43 – 84 × 40 mm).

#### Synonymy.

See Powell (1951).

#### Remarks.

Cárdenas et al. (2008) explained that some authors considered *Fusitriton cancellatus* (Lamark, 1816) as a valid synonym. Concurringly, Zelaya (2009a) reported *Fusitriton magellanicus* as a synonym of *Fusitriton cancellatus*. However, according to Bouchet (2012), the taxonomically accepted name of the species is *Fusitriton magellanicus*.

#### Distribution.

WTSP: from Los Vilos to Valparaíso (McLean and Andrade 1982). Magellanic: Gulf of Ancud (Cárdenas et al. 2008), Chiloé Archipelago (USNM 2010), Gulf of Corcovado (Cárdenas et al. 2008), Puerto Cóndor (Strebel 1905b), and Smyth Channel (Strebel 1905b); Strait of Magellan (Húpe 1854, Tryon 1881): Río Seco (Strebel 1905b), Punta Arenas (Strebel 1905b), Punta Santa Ana (this record), and Carlos III Island (Aldea et al. 2011a); Beagle Channel (Strebel 1905b), Orange Bay (Rochebrune and Mabille 1889), and Cape Horn (Powell 1951); Malvinas/Falkland Islands (Powell 1951), and Le Maire Strait (Strebel 1905b). WTSA: from Río Grande do Sul (Smith 1970), and Uruguay (Scarabino 2004). SO: Bellingshausen Sea (USNM 2010). Other sites: South Africa (OBIS 2014), Australia (Rosenberg et al. 2002), and New Zealand (Stocks 2003).

### Eatoniella nigra

Taxon classificationAnimaliaLittorinimorphaEatoniellidae

(d’Orbigny, 1840)

Fig. 5F

#### Material examined.

1 spm (2 × 1 mm).

#### Synonymy.

See Ponder and Worsfold (1994).

#### Remarks.

It was described under the name *Paludestrina nigra* d’Orbigny, 1840 for the north of Chile. Afterwards, Marincovich (1973) described the species *Eatoniella latina* being the first representative Eatoniellidae for the Southeast Pacific. However, Ponder and Worsfold (1994), upon revising the shells of both species, found a common morphology, lower, more ovular, and thinner than the other black-colored species present in South America. Thus, *Eatoniella latina* is considered a junior synonym of this species. Records from South Africa (Rosenberg et al. 2002, OBIS 2014) likely correspond to *Eatoniella afronigra* according to Ponder and Worsfold (1994).

#### Distribution.

WTSP: Iquique (Marincovich 1973, Ponder and Worsfold 1994), and Antofagasta (Ponder and Worsfold 1994). Magellanic: Puerto Montt (Ponder and Worsfold 1994), Chiloé Archipelago (Ponder and Worsfold 1994), and Coyhaique (Ponder and Worsfold 1994); Strait of Magellan: western entrance of the Strait of Magellan (Ponder and Worsfold 1994) and Punta Santa María (this record); Staten Island (Ponder and Worsfold 1994).

### Eumetula pulla

Taxon classificationAnimaliaCaenogastropodaNewtoniellidae

(Philippi, 1845)

Fig. 5G

#### Material examined.

2 spm (3 × 1 – 7 × 3 mm).

#### Synonymy.

See Cárdenas et al. (2008).

#### Remarks.

Cárdenas et al. (2008) noted that this species is different from the other species in its family because it does not have cords on the base. Morris and Rosenberg (2005) considered *Cerithium caelatum* (Gould, 1849) as a synonym of *Eumetula pulla*. However, Zelaya (2009a) considered it a valid species and suggested a significant revision of this complex of species.

#### Distribution.

Magellanic: Gulf of Corcovado (Cárdenas et al. 2008), Estero Elefantes (Reid and Osorio 2000), and Smyth Channel (Strebel 1905b); Strait of Magellan (Strebel 1905b): Punta Santa María (this record), Punta Santa Ana (this record), Cape Valentín (Strebel 1905b), Dawson Island (Strebel 1905b), Puerto Cóndor, Inútil Bay (Strebel 1905b), and Carlos III Island (Aldea et al. 2011a); Beagle Channel (Strebel 1908), Navarino Island (Strebel 1905b), and Puerto Toro (Strebel 1905b); Malvinas/Falkland Islands (Strebel 1905b, 1908, Powell 1951), Burdwood Bank (Melvill and Standen 1912), and Le Maire Strait (Strebel 1905b). WTSA: Río de la Plata (Carcelles and Williamson 1951), and Mar del Plata (Castellanos 1971).

### Savatieria meridionalis

Taxon classificationAnimaliaNeogastropodaBuccinidae

(Smith, 1881)

Fig. 5H

#### Material examined.

1 spm (5 × 2 mm).

#### Synonymy.

See Dell (1972).

#### Remarks.

Dell (1972) explained that seven species of the genus *Savatieria* have been described for the Magellan Region and the Malvinas/Falkland Islands. However, this genus is not well studied, and the Magellanic species are slightly different and not well represented in collections (Dell 1972).

#### Distribution.

Magellanic: Strait of Magellan (Smith 1881): Punta Santa María (this record) and Carlos III Island (Aldea et al. 2011a) and Cape Valentín (Strebel 1905b); Beagle Channel (Osorio 1999), Fortescue Bay (Strebel 1905b), Puerto Angosto (Strebel 1905b), Basket Island (Strebel 1905b), Puerto Eugenia (Strebel 1905b), and Picton Island (Strebel 1905b); Santa Cruz (Castellanos 1979) and Malvinas/Falkland Islands (Strebel 1905b, 1908) in Port Stanley (Strebel 1905b).

### Pareuthria cerealis

Taxon classificationAnimaliaNeogastropodaBuccinidae

(Rochebrune & Mabille, 1885)

Fig. 6A

#### Material examined.

6 spm (5 × 2 – 6 × 3).

#### Synonymy.

See Cárdenas et al. (2008).

#### Remarks.

This species presents a ruddy-yellow coloring, and one of its most distinguishable characteristics is its smooth texture with one or two stripes under the sutures of each whorl (Cárdenas et al. 2008). Our specimens presented quite eroded shells.

#### Distribution.

Magellanic: Gulf of Ancud (Cárdenas et al. 2008); Strait of Magellan: Punta Santa María (this record) and Carlos III Island (Aldea et al. 2011a); Beagle Channel (Rochebrune and Mabille 1889), Orange Bay (Rochebrune and Mabille 1889), Oglander Bay (Linse 2002), Goree Passage (Linse 2002), and Picton Island (Linse 2002); from 47°S in South Atlantic Ocean (Morris and Rosenberg 2005), and Malvinas/Falkland Islands (Castellanos and Landoni 1993) in Port Stanley (Strebel 1905).

**Figure 6.**
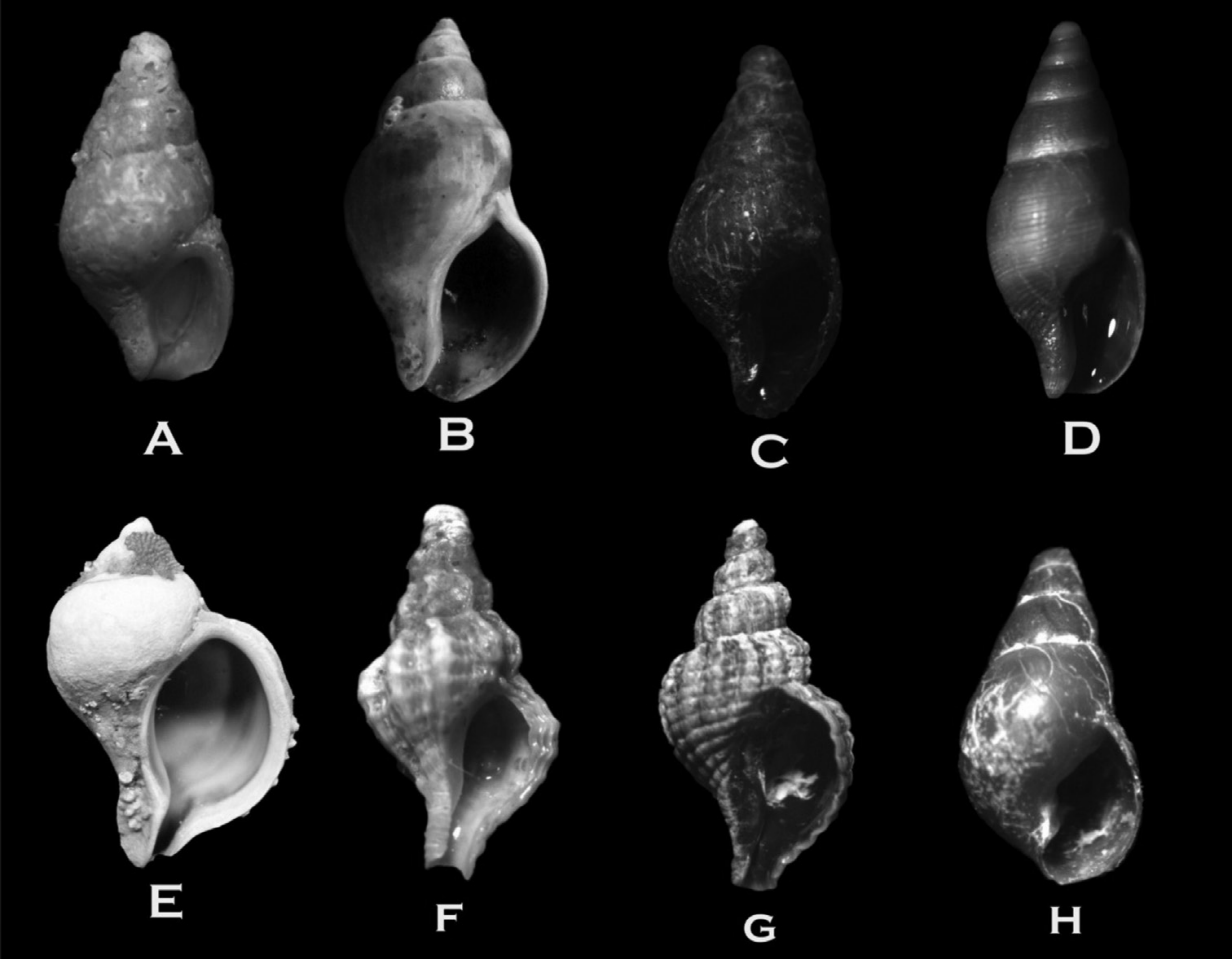
**A** *Pareuthria cerealis* (6 × 3 mm) **B** *Pareuthria plumbea* (25 × 12 mm) **C** *Pareuthria paessleri* (7 × 4 mm) **D** *Pareuthria janseni* (10 × 14 mm) **E** *Trophon gerversianus* (53 × 38 mm) **F** *Fuegotrophon pallidus* (18 × 9 mm) **G** *Xymenopsis muriciformis* (32 × 17 mm) **H** *Acteon biplicatus* (5 × 3 mm).

### Pareuthria plumbea

Taxon classificationAnimaliaNeogastropodaBuccinidae

(Philipi, 1844)

Fig. 6B

#### Material examined.

20 spm (13 × 6 – 25 × 12 mm).

#### Synonymy.

See Dell (1971).

#### Remarks.

Aldea and Rosenfeld (2011) commented that, differing from other species of the family Buccinidae, it is characterized by direct development during its reproductive cycle by depositing egg masses (Pastorino and Penchaszadeh 2002). Dell (1971) explained that Strebel (1905b), when revising the species of the genus *Pareuthria*, observed a great similarity between *Pareuthria plumbea* and *Pareuthria magellanica*; however, the original figures did not concur with the distinction that was made by Strebel (1905b).

#### Distribution.

Magellanic: Puerto Edén, Levinson Island, Paso de Indio, and Piazzi Island (Dell 1971); Strait of Magellan (Powell 1951): Buque Quemado (Aldea and Rosenfeld 2011), Laredo Bay (Mutschke et al. 1998, Linse 2002), Punta Arenas (USNM 2010), Punta Santa Ana (Ríos et al. 2007; this record), Cape Froward (USNM 2010), Dawson Island (USNM 2010), and Carlos III Island (Aldea et al. 2011a); London Island (Pelseneer 1903), Beagle Channel (Pelseneer 1903), Ushuaia (Strebel 1908), Puerto Williams (Dell 1971), Róbalo Bay (Dell 1971, Ojeda et al. 2010), Hermite Islands (Dell 1971), Bertrand Island (Dell 1971), and Seno Grandi (Dell 1971); Malvinas/Falkand Islands (Dell 1971, USNM 2010), San Sebastián Bay (USNM 2010), Buen Suceso Bay (USNM 2010), and Staten Island (USNM 2010). WTSA: from 38°S toward south (Morris and Rosenberg 2005).

### Pareuthria paessleri

Taxon classificationAnimaliaNeogastropodaBuccinidae

(Strebel, 1905)

Fig. 6C

#### Material examined.

1 spm (7 × 4 mm).

#### Synonymy.

See Powell (1951).

#### Remarks.

This species is similar to the species *Pareuthria cerealis* but differs in that the last whorl is more globular, and it has spiral stripes in the base of the last whorl (Cárdenas et al. 2008). Our examined specimen presented an eroded shell.

#### Distribution.

Magellanic: Smyth Channel (Powell 1951); Strait of Magellan (USNM 2010): Punta Santa María (this record) and Carlos III Island (Aldea et al. 2011a); Ushuaia (Strebel 1905b) and Puerto Eugenia (Strebel 1905b); Le Maire Strait (Strebel 1905b).

### Pareuthria janseni

Taxon classificationAnimaliaNeogastropodaBuccinidae

(Strebel, 1905)

Fig. 6D

#### Material examined.

1 spm (10 × 14 mm).

#### Synonymy.

*Euthria janseni* Strebel 1905b: 622, pl. 21, fig. 7-7a; Strebel 1908: 28.

*Pareuthria janseni*, Forcelli 2000: 96, fig. 265.

#### Remarks.

The specimen analyzed in this study had light spiral stripes on the whole surface of the shell, which is characteristic of this species. Similarly, Strebel (1905b) commented that the last whorl presented 30 spiral stripes. This species is very similar to the species *Pareuthria michaelseni*, but it can be distinguished by a more globular last whorl, occupying ¾ of the total shell length (Forcelli 2000).

#### Distribution.

Magellanic: eastern micro-basin of the Strait of Magellan (Ríos et al. 2003), Punta Santa Ana (this record), Ushuaia (Strebel 1905b), Beagle Channel (Osorio 1999), Picton Island (Strebel 1905b), and Cape Horn (Osorio 1999); Puerto Deseado (Forcelli 2000) and Malvinas/Falkland Islands (Strebel 1908).

### Trophon geversianus

Taxon classificationAnimaliaNeogastropodaMuricidae

(Pallas, 1774)

Fig. 6E

#### Material examined.

77 spm (30 × 17 – 53 × 38 mm).

#### Synonymy.

See Pastorino (2005b).

#### Remarks.

*Trophon geversianus* is the most well-known species of the genus *Trophon*. Its morphological variability is evident in the quantity of names proposed for each morphotype of this species (Pastorino 2005b). The rest of the nominal species from the Southern Ocean and adjacent waters displaying a similar morphology were compared by Aldea and Troncoso (2010a).

#### Distribution.

Magellanic: Strait of Magellan (Rochebrune and Mabille 1889, Lamy 1906b, Powell 1951, Houart 1998, Mutschke et al. 1998, Osorio 1999, Linse 2002, Pastorino 2005b, USNM 2010, Aldea et al. 2011a): Punta Santa María (this record); Usuahia (Rochebrune and Mabille 1889, Strebel 1908, Pastorino 2005b), Beagle Channel (Rochebrune and Mabille 1889, Pelseneer 1903, Dell 1971, Osorio 1999), Róbalo Bay (Ojeda et al. 2010), and Cape Horn (Rochebrune and Mabille 1889); San Antonio Oeste, Sierra Grande, Puerto Lobos, Puerto Pirámides, Puerto Madryn, San Jorge Gulf, Puerto Deseado, and Punta Peñas (Pastorino 2005b), Santa Cruz River (Powell 1951, Pastorino 2005b), Río Gallegos (Pastorino 2005b), Malvinas/Falkland Islands (Watson 1886, Melvill and Standen 1898, Melvill and Standen 1907, Strebel 1908, Powell 1951, Pastorino 2005b), Burdwood Bank (Melvill and Standen 1912, Pastorino 2005b), and Staten Island (USNM 2010). WTSA: Buenos Aires (Morris and Rosenberg 2005). SO: Heard Island (Watson 1886); records from western Antarctic Peninsula (Lamy 1906b) could correspond to an Antarctic species (Aldea and Troncoso 2010a).

### Fuegotrophon pallidus

Taxon classificationAnimaliaNeogastropodaMuricidae

(Broderip, 1833)

Fig. 6F

#### Material examined.

3 spm (6 × 3 – 18 × 9 mm).

#### Synonymy.

See Houart (2010).

#### Remarks.

The species that was referred to under the genus *Fuegotrophon* by Pastorino (2002) that was originally proposed as a subgenus by Powell (1951) based principally on the characteristics of the protoconch and radula. Currently, the name *Fuegotrophon pallidus* is considered to represent a separate genus (Houart 2010).

#### Distribution.

Magellanic: Gulf of Ancud and Gulf of Corcovado (Cárdenas et al. 2008); Strait of Magellan (Powell 1951, Mutschke et al. 1998, Osorio 1999, Linse 2002): Desolación Island (USNM 2010), Punta Santa María, and Punta Santa Ana (this record); Beagle Channel (Osorio 1999, Linse 2002) and Cape Horn (Rochebrune and Mabille 1889, Linse 2002, USNM 2010); Malvinas/Falkland Islands (Melvill and Standen 1907, Powell 1951) and Burdwood Bank (Melvill and Standen 1907, Strebel 1908, USNM 2010). WTSA: from 38°S toward south (Morris and Rosenberg 2005). SO: Drake Passage (Powell 1951) and Crozet Island (Cantera and Arnaud 1985).

### Xymenopsis muriciformis

Taxon classificationAnimaliaNeogastropodaMuricidae

(King & Broderip, 1832)

Fig. 6G

#### Material examined.

51 spm (5 × 3 – 32 × 17 mm).

#### Synonymy.

See Pastorino and Harasewych (2000).

#### Remarks.

This species has a similar morphology to *Xymenopsis subnodosus* (Gray, 1839) in that it presents an external crenulate margin of the aperture, 12–16 axial cords on the last whorl, and 22–24 spiral cords (Pastorino and Harasewych 2000). *Xymenopsis muriciformis* has a direct development during its reproductive cycle, depositing its egg masses on rocky substrates (Zelaya 2009a).

#### Distribution.

Magellanic: Chonos Archipelago (Húpe 1854, Rochebrune and Mabille 1889), Puerto Edén (Dell 1971), Traiguén Island (Reid and Osorio 2000), Guarello Island (Pastorino and Harasewych 2000), Paso de Indio (Dell 1971), Madre de Dios Island (Pastorino and Harasewych 2000), and Smyth Channel (Strebel 1904); Strait of Magellan (King and Broderip 1832, Húpe 1854, Tryon 1880, Rochebrune and Mabille 1889, Strebel 1904, Powell 1951, Pastorino and Harasewych 2000): Punta Arenas (Strebel 1904, Pastorino and Harasewych 2000), Punta Santa María (this record), Gente Grande Bay (Strebel 1904), Inútil Bay (Strebel 1904, USNM 2010), Puerto del Hambre (Pastorino and Harasewych 2000), Punta Santa Ana (Ríos et al. 2007; this record), Cape Froward (USNM 2010), Dawson Island (Pastorino and Harasewych 2000, USNM 2010), and Carlos III Island (Pastorino and Harasewych 2000, Aldea et al. 2011a); Cockburn Channel (Pastorino and Harasewych 2000), Ushuaia (Strebel 1904, Pastorino and Harasewych 2000), Puerto Harberton (Strebel 1904, Pastorino and Harasewych 2000), Beagle Channel (Pastorino and Harasewych 2000), Navarino Island (Strebel 1904), Puerto Williams (Dell 1971), Orange Bay (Tryon 1880, Rochebrune and Mabille 1889, Lamy 1906b), and Cape Horn (Strebel 1904); from 41°S toward south in the South Atlantic Ocean (Morris and Rosenberg 2005), Puerto Deseado (Pastorino and Harasewych 2000), Tierra del Fuego (Pastorino and Harasewych 2000), San Sebastián Bay (Pastorino and Harasewych 2000, USNM 2010), Malvinas/Falkland Islands (Watson 1886, Powell 1951, Castellanos and Landoni 1993, Pastorino and Harasewych 2000, USNM 2010), Cape Buen Tiempo (Pastorino and Harasewych 2000), Port Stanley (Strebel 1904), Lively Island (Pastorino and Harasewych 2000), Staten Island (Pastorino and Harasewych 2000), Puerto Año Nuevo (Pastorino and Harasewych 2000), and Cape San Diego (USNM 2010).

### Acteon biplicatus

Taxon classificationAnimaliaNot assignedActeonidae

(Strebel, 1908)

Fig. 6H

#### Material examined.

2 spm (4 × 1.5 – 5 × 3 mm).

#### Synonymy.

*Odostomia biplicata* Strebel 1908: 65, pl. I, fig. 9a.

*Acteon biplicata*, Castellanos and Landoni 1986: 297, pl. I, fig. 9.

*Acteon biplicatus*, Castellanos et al. 1993: 7, pl. I, fig. 3; Forcelli 2000: 115, fig. 347; Cárdenas et al. 2008: 223, fig. 5.56; Aldea et al. 2011b: 43, fig. 3B.

#### Remarks.

The morphology of this species is similar to *Acteon elongatus* Castellanos, Rolán & Bartolotta, 1987. However, it can be differentiated because *Acteon elongatus* does not have a columellar tooth and has a wider aperture (Aldea et al. 2011b).

#### Distribution.

Magellanic: Coldita Channel (Cárdenas et al. 2008), Messier Channel, and Wide Channel (Aldea et al. 2011b); Strait of Magellan: eastern micro-basin of the Strait of Magellan (Ríos et al. 2003) and Punta Santa María (this record). South Atlantic Ocean: from 43°S (Morris and Rosenberg 2005), Malvinas/Falkland Islands (Castellanos et al. 1993), and Berkeley Sound (Strebel 1908).

### Aulacomya atra

Taxon classificationAnimaliaMytiloidaMytilidae

(Molina, 1782)

Fig. 7A

#### Material examined.

3 spm (8 × 4 – 14 × 7 mm).

#### Synonymy.

See Reid and Osorio (2000).

#### Remarks.

Reid and Osorio (2000) noted that this species is easily distinguishable from the other species of mytilids that exist on the Chilean coast, given its radial ribs on valves. However, specimens less than 40 mm could be confused with *Perumytilus purpuratus* (Lamark, 1819). But at that size, *Aulacomya atra* presents a yellowish or ruddy color, while *Perumytilus purpuratus* has a black periostracum and double the radial ribs (Reid and Osorio 2000).

#### Distribution.

WTSP: Callao in Perú (Dall 1909, Soot-Ryen 1955), Iquique (Marincovich 1973), Antofagasta (Guzman et al. 1998), Coquimbo (Húpe 1854, Carcelles 1950), from Punta Pingueral to Mocha Island (Aldea and Valdovinos 2005), and Valdivia (Zagal and Hermosilla 2001). Magellanic: Coldita Channel (Cárdenas et al. 2008), Estero Elefantes (Reid and Osorio 2000), Puerto Edén (Dell 1971), Levinson Island (Dell 1971), and Piazzi Island (Dell 1971); Strait of Magellan (Húpe 1854, Rochebrune and Mabille 1889, USNM 2010): eastern micro-basin of the Strait of Magellan (Ríos et al. 2003), Laredo Bay (Mutschke et al. 1998), Punta Santa María (this record), Inútil Bay (USNM 2010), Punta Santa Ana (Ríos et al. 2007; this record), Cape Froward (USNM 2010), Dawson Island (USNM 2010), Carlos III Island (Aldea et al. 2011a), and Desolación Island (USNM 2010); Beagle Channel (Pelseneer 1903), Puerto Williams (Dell 1971), Róbalo Bay (Dell 1971, Ojeda et al. 2010), Puerto Toro (Pelseneer 1903), Hermite Islands (Dell 1971), Bertrand Island (Dell 1971), and Orange Bay (Rochebrune and Mabille 1889, Lamy 1906a); San José Gulf (Zaixo 2004), Cape Penas (USNM 2010), San Sebastián Bay (USNM 2010), Malvinas/Falkland Islands (Húpe 1854, Melvill and Standen 1907, Dell 1964, USNM 2010), and Staten Island (USNM 2010). WTSA: southern Brazil (Soot-Ryen 1955), Uruguay (Scarabino 2003b), and Puerto Quequén (Carcelles 1944). SO: Scotia Sea (USNM 2010), and Kerguelen Islands (Lamy 1906a, Carcelles 1950, Troncoso et al. 2001). Other sites: South Africa (Huber 2013), and New Zealand. Northern Hemisphere: North Sea (Huber 2013).

**Figure 7.**
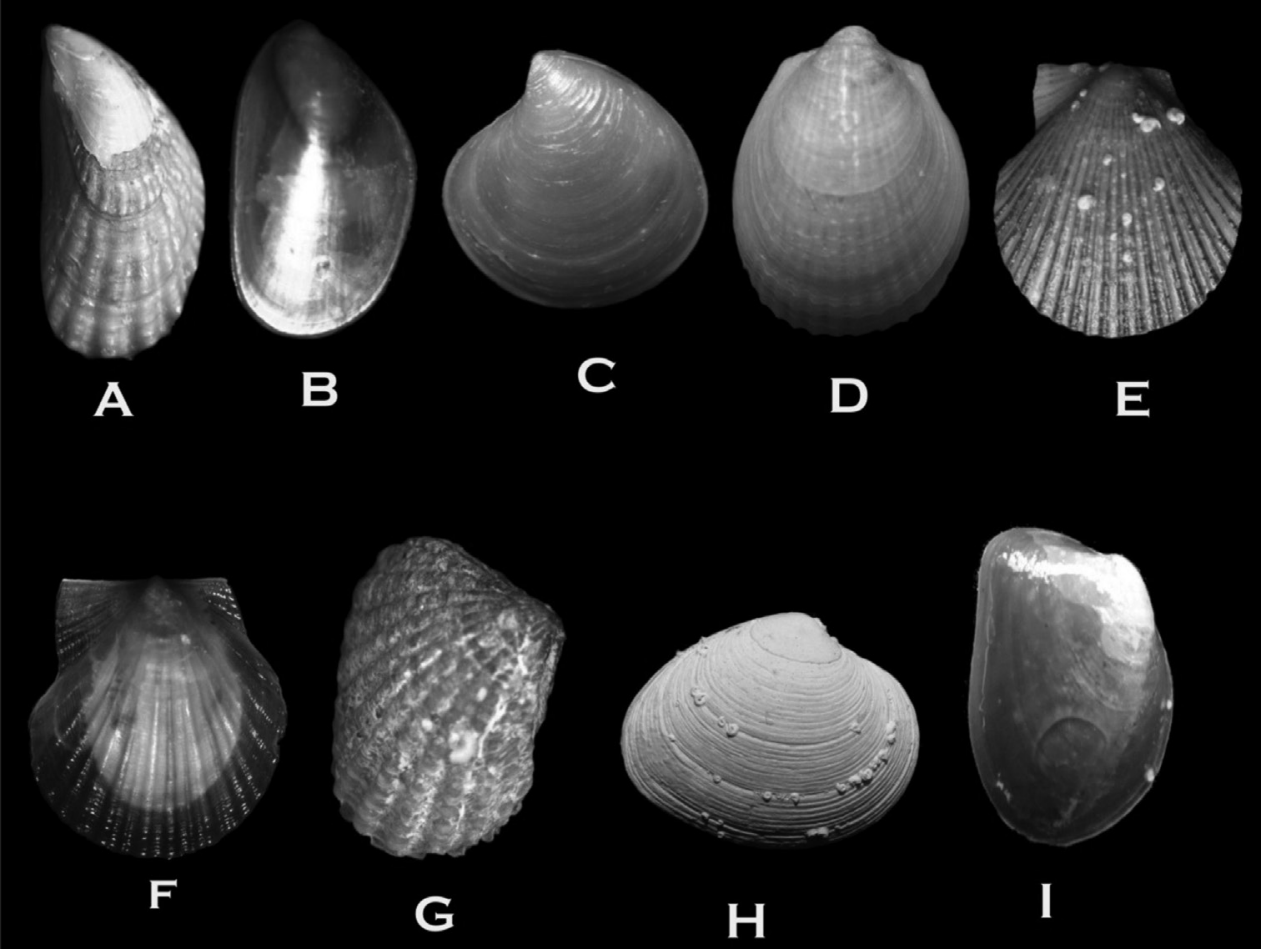
**A** *Aulacomya atra* (8 × 4 mm) **B** *Mytilus edulis chilensis* (4 × 3 mm) **C** *Astarte longirostra* (5 × 5 mm) **D** *Limea pygmaea* (9 × 6 mm) **E** *Zygochlamys patagonica* (14 × 11 mm) **F** *Austrochlamys natans* (7.1 × 6.5 mm) **G** *Carditella naviformis* (5 × 3.5 mm) **H** *Tawera elliptica* (10 × 11 mm) **I** *Gaimardia trapesina* (14 × 7 mm).

### Mytilus edulis platensis

Taxon classificationAnimaliaMytiloidaMytilidae

(d’Orbigny, 1842)

Fig. 7B

#### Material examined.

1 spm (4 × 3 mm).

#### Synonymy.

See Reid and Osorio (2000).

#### Remarks.

Regarding the current status of this species, Aldea and Rosenfeld (2011) commented that in spite of the genetic and morphological study carried out by Toro (1998), who placed this species in *Mytilus edulis chilensis*, the taxonomic problem is still not resolved. The study carried out by Cárcamo et al. (2005) on specimens from the Chilean Coast was based on allozymes and compared these specimens with European specimens of *Mytilus edulis* and *Mytilus galloprovincialis* (Lamark, 1819). The authors concluded that the Chilean specimens should rather be considered a subspecies of *Mytilus galloprovincialis* given that it is genetically closer to this species, but having particular and characteristic allele frequencies. Investigating the taxonomy and genetics of Chilean smooth-shelled *Mytilus*, Borsa et al. (2012) concluded that *Mytilus edulis* from the northern hemisphere is different from *Mytilus edulis* from the southern hemisphere in proportion to the nuclear loci and the mitochondrial locus. For this reason they consider them as geographically isolated entities. Thus, the Chilean Blue mussles are considered to represent subspecies of *Mytilus edulis*. Following the principle of priority, the authors stress that *platensis* d’Orbigny, 1842 is the correct subspecific name for the southern hemisphere *Mytilus edulis*, and relegate the name *Mytilus chilensis* Hupé, 1854 into the synonymy of *platensis*. Larrain et al. (2012) applied the Me 15–16 marker to samples from sites between 41°S and 51°S and found that the majority of the mussels corresponded to “*Mytilus chilensis*”, and saw no evidence for an occurrence of *Mytilus edulis*. Additionally, putative hybrids of *Mytilus chilensis* × *Mytilus trossulus* and *Mytilus chilensis* × *Mytilus galloprovincialis* were detected, and the authors stressed that other markers are needed to differentiate between the southern hemisphere *Mytilus* species. Concluding it can be said the the correct taxonomic allocation for the southern-hemisphere *Mytilus* species is still pending. For the time being, we here use the name *platensis* d’Orbigny, 1842 as a subspecies of *Mytilus edulis* for the specimens from our samples.

#### Distribution.

WTSP: Iquique (Soot-Ryen 1959), Valparaíso (Húpe 1854, Dall 1909), from Punta Pingueral to Mocha Island (Aldea and Valdovinos 2005), and Valdivia (Zagal and Hermosilla 2001, Borsa et al. 2012). Magellanic: Puerto Montt (Borsa et al. 2012), Calbuco (Borsa et al. 2012), Gulf of Ancud (Cárdenas et al. 2008), Estero Elefantes (Reid and Osorio 2000), Estero Castro (Dell 1971), Puerto Edén (Dell 1971), and Piazzi Island (Dell 1971); Strait of Magellan (Rochebrune and Mabille 1889, Dell 1964): eastern micro-basin of the Strait of Magellan (Ríos et al. 2003), Buque Quemado (Aldea and Rosenfeld 2011), Laredo Bay (Mutschke et al. 1998), Punta Santa Ana (Ríos et al. 2007), Punta Santa María (this record), Cape Froward (USNM 2010), Dawson Island (USNM 2010), and Carlos III Island (Aldea et al. 2011a); Puerto Williams (Dell 1971), Róbalo Bay (Dell 1971, Ojeda et al. 2010), Hermite Islands (Dell 1971), Bertrand Island (Dell 1971), Seno Grandi (Dell 1971), and Orange Bay (Rochebrune and Mabille 1889); Chubut (Carcelles 1944), Malvinas/Falkland Islands (Dell 1964), San Sebastián Bay (USNM 2010), and Staten Island (USNM 2010). WTSA: Uruguay (Scarabino 2003b), and Buenos Aires Province (Carcelles 1944).

### Astarte longirostra

Taxon classificationAnimaliaCarditoidaAstartidae

(d’Orbigny, 1842)

Fig. 7C

#### Material examined.

4 spm (4.5 × 4 – 5 × 5 mm).

#### Synonymy.

See Dell (1964).

#### Remarks.

Dell (1990) explained that this is the only species from the genus in the Magellan Region, given that the species *Astarte magallenica* (Smith, 1881) constitutes a morphological variation of *Astarte longirostra* (Dell 1964).

#### Distribution.

Magellanic: Strait of Magellan (Smith 1881, USNM 2010): eastern micro-basin of the Strait of Magellan (Ríos et al. 2003), Tierra del Fuego (Dell 1964), Punta Santa María (this record), and Carlos III Island (Aldea et al. 2011a); Hoste Island (USNM 2010), Beagle Channel (Rochebrune and Mabille 1889), and Cape Horn (USNM 2010); from 45°S toward south in the South Atlantic Ocean (Bigatti 2010), Malvinas/Falkland Islands (Dell 1964, Hain 1990), Le Maire Strait (USNM 2010), and Staten Island (USNM 2010). SO: Marion Island (Hain 1990), Prince Edward Island (Smith 1881), Kerguelen Islands (Powell 1960, Hain 1990), South Georgia Island (Powell 1960, Hain 1990, USNM 2010), South Shetland Islands (Hain 1990, USNM 2010), Ross Sea (USNM 2010), and Weddell Sea (Gutt et al. 2000).

### Limea pygmaea

Taxon classificationAnimaliaLimoidaLimidae

(Philippi, 1845)

Fig. 7D

#### Material examined.

4 spm (4 × 2.5 – 9 × 6 mm).

#### Synonymy.

See Aldea and Troncoso (2008).

#### Remarks.

Aldea and Troncoso (2010b) commented that this species is similar to *Limatula ovalis* (Thiele, 1912) but smaller and thinner. Both species present direct development through incubation (Linse and Page 2003).

#### Distribution.

Magellanic: Smyth Channel (Thiele 1912); Strait of Magellan, (Húpe 1854, Lamy 1906a, Dell 1990): eastern micro-basin of the Strait of Magellan (Ríos et al. 2003), Punta Santa María (this record), and Carlos III Island (Aldea et al. 2011a); Orange Bay (Rochebrune and Mabille 1889); Malvinas/Falkland Islands (Dell 1964, Linse 1997), and Staten Island (Dell 1990). WTSA: Uruguay (Scarabino 2003b), and Buenos Aires Province (Carcelles 1944). SO: South Shetland Islands (Dell 1990, Narchi et al. 2002, Aldea and Troncoso 2008), Macquaire Island (Powell 1960), Kerguelen Islands (Smith 1879, Smith 1885, Thiele 1912, Thiele and Jaeckel 1931, Powell 1957, Troncoso et al. 2001), Marion and Prince Edward Islands (Smith 1885, Branch et al. 1991).

### Zygochlamys patagonica

Taxon classificationAnimaliaPectinoidaPectinidae

(King & Broderip, 1832)

Fig. 7E

#### Material examined.

2 spm (12 × 10 – 14 × 11 mm).

#### Synonymy.

See Cárdenas et al. (2008).

#### Remarks.

Waloszek (1984) reported that the species has a wide variability in its morphological characteristics, presenting different types of sculpture. Coloration can range from white to dark red and yellow. This species is found in shallow waters and on the front of slopes, where it forms large banks (Zelaya 2009b).

#### Distribution.

Magellanic: Chiloé Archipelago (Waloszek 1984), Estero Elefantes (Reid and Osorio 2000), and Wellington Island in Puerto Edén (Dell 1971); Strait of Magellan (King and Broderip 1832): eastern micro-basin of the Strait of Magellan (Ríos et al. 2003), Carlos III Island (Aldea et al. 2011a), Punta Santa Ana (Ríos et al. 2007), and Punta Santa María (this record); Beagle Channel (Rochebrune and Mabille 1889) and Cape Horn (Waloszek 1984); Chubut (Carcelles 1944), Santa Cruz (Carcelles 1944), and Malvinas/Falkland Islands (Grau 1959) toward 55°S (Morris and Rosenberg 2005). WTSA: Uruguay (Scarabino 2003b), and Buenos Aires Province (Carcelles 1944). SO: South Shetland Islands (USNM 2010).

### Austrochlamys natans

Taxon classificationAnimaliaPectinoidaPectinidae

(Philippi, 1845)

Fig. 7F

#### Material examined.

1 spm (7.1 × 6.5 mm).

#### Synonymy.

See Dell (1971).

#### Remarks.

Dell (1971) concluded that this species inhabits fronds of the giant kelp *Macrocystis pyrifera* and that juveniles present a thin shell that is semitransparent, due to an adaptation to this environment. In relation to its comparative morphology, it can be differentiated from *Zygochlamys patagonica* because of its globular, delicate shell and wider radial cords (Zelaya 2009b).

#### Distribution.

Magellanic: Punta Gaviota and Carlos Island (Dell 1971); Strait of Magellan (King and Broderip 1832): Punta Santa Ana (this record), Dawson Island (USNM 2010), and Carlos III Island (Aldea et al. 2011a); London Island (Pelseneer 1903) and Puerto Williams (Dell 1971).

### Carditella naviformis

Taxon classificationAnimaliaCarditoidaCondylocardiidae

(Reeve, 1843)

Fig. 7G

#### Material examined.

13 spm (4 × 2 – 5 × 3.5 mm).

#### Synonymy.

See Güller and Zelaya (2013).

#### Remarks.

This species is very similar to *Carditella tegulata* (Reeve, 1843), which has a triangular contour, but its shell is equilateral, with a central umbo and straight upper and lower dorsal margins (Zelaya 2009b). Accordingly, Smith (1881) distinguished the species due to the presence of 14–15 radial ribs and a central umbo. However, the specimens revised by Reid and Osorio (2000) had a corresponding sculpture to *Carditella naviformis*, but the radial ribs were slightly pronounced from 11 to 13 in number, and the margins of the shell were more similar to *Carditella tegulata*.

#### Distribution.

WTSP: Iquique and Tocopilla (Soot-Ryen 1959), and Valparaíso (Hupé 1854, Dall 1903, Ramorino 1968, Güller and Zelaya 2013). Magellanic: Gulf of Ancud, Comau Fjord and Gulf of Corcovado (Güller and Zelaya 2013), Darwin Channel (Güller and Zelaya 2013), and Estero Elefantes (Reid and Osorio 2000); Strait of Magellan (Carcelles and Williamson 1951, USNM 2010): Carlos III Island (Aldea et al. 2011a) and Punta Santa María (this record); Cockburn Channel (Güller and Zelaya 2013), Beagle Channel (Güller and Zelaya 2013), and Cape Horn (USNM 2010); Malvinas/Falkland Islands (Melvill and Standen 1914), Staten Island (USNM 2010, Güller and Zelaya 2013), and Le Maire Strait (USNM 2010).

### Tawera elliptica

Taxon classificationAnimaliaVeneroidaVeneridae

(Lamark, 1818)

Fig. 7H

#### Material examined.

9 spm (8 × 10 – 10 × 11 mm).

#### Synonymy.

See Gordillo (2006).

#### Remarks.

The morphology of this species is similar to the smallest specimens of *Retrotapes exalbidus*. Zelaya (2009b) showed that they can be differentiated in that *Tawera elliptica* has wider cords and finer interspaces and the inside of the shell is either purplish or brownish. All specimens collected during this study had a strong violet coloring on the inside of the valves.

#### Distribution.

WTSP: Valparaíso (Húpe 1854, Osorio and Bahamonde 1970). Magellanic: Gulf of Corcovado (Cárdenas et al. 2008), and Traiguén Island (Reid and Osorio 2000); Strait of Magellan (USNM 2010): Punta Santa María (this record), Dawson Island (USNM 2010), and Carlos III Island (Aldea et al. 2011a); Beagle Channel (Rochebrune and Mabille 1889), Ushuaia (USNM 2010), Puerto Williams (Dell 1971), Orange Bay (Rochebrune and Mabille 1889), and Cape Horn (USNM 2010); Malvinas/Falkand Islands (Dell 1964, Linse 1997), and Staten Island (USNM 2010). WTSA: Río Grande do Sul and Uruguay (Gordillo 2006), and Buenos Aires Province (Carcelles 1944).

### Gaimardia trapesina

Taxon classificationAnimaliaVeneroidaGaimardiidae

(Lamarck, 1819)

Fig. 7I

#### Material examined.

3 spm (14 × 7 – 14 × 22 mm).

#### Synonymy.

See Morris and Rosenberg (2005).

#### Remarks.

This species is an epibiont of the giant kelp *Macrocystis pyrifera* (Ralph and Maxwell 1977), although it can also be found in blocks and by personal observation. It is an incubating species that retains embryos in the gills until they are completely developed.

#### Distribution.

Magellanic: Strait of Magellan (Hombron and Jacquinot 1854): Punta Santa María (this record), Carlos III Island (Aldea et al. 2011a), and Fuerte Bulnes (pers. obs.); Orange Bay (Rochebrune and Mabille 1889); Malvinas/Falkland Islands (Melvill and Standen 1907, USNM 2010), and Staten Island (USNM 2010). WTSA: Rio Grande do Sul (Morris and Rosenberg 2005, Dias Passos and Magalhães 2011), Uruguay (Scarabino 2003b), and Buenos Aires Province (Carcelles 1944). SO: South Georgia Island (Martens and Pfeffer 1886).

## Biogeography

**Figure 8.**
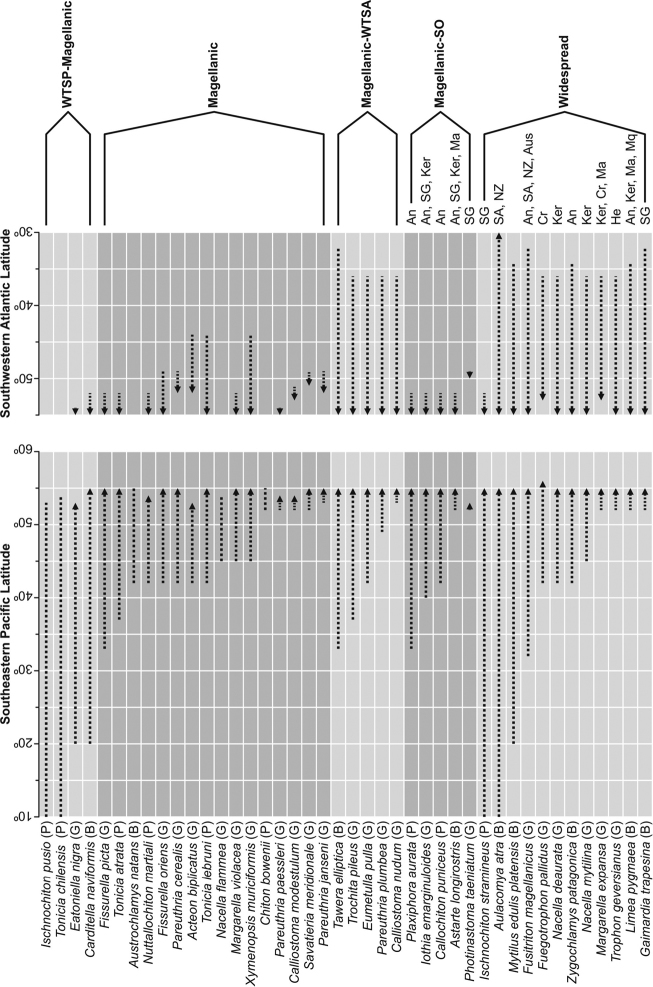
Biogeographic distribution of molluscs associated with natural beds of *Gigartina skottsbergii* in the Strait of Magellan. Biogeographical provinces were taken from Spalding et al. (2007): Warm Temperate South-eastern Pacific (WTSP), Warm Temperate South-western Atlantic (WTSA), and Southern Ocean (SO). Antarctica (An), South Georgia (SG), Kerguelen Island (Ker), Marion Island (Ma), South Africa (SA), New Zealand (NZ), Australia (Aus), Crozet Island (Cr), Heard Island (He), and Macquaire Island (Mq). (P) indicates Polyplacophora, (G) Gastropoda and (B) Bivalvia.

Of the identified 42 species, 29% have a wide distribution, 9% are distributed in the Warm Temperate South-eastern Pacific-Magellanic provinces, 38% are Magellanic (*sensu stricto*), and 12% present a Warm Temperate Southwestern Atlantic-Magellanic distribution and Magellanic-Southern Ocean distribution, respectively (Fig. 8).

Taking into account the 9 species of the class Polyplacophora recorded in this study, only the species *Callochiton puniceus* and *Plaxiphora aurata* showed a Magellanic-Southern Ocean distribution, while two species were found in the Southeast Temperate Magellanic-Pacific area and four species were distributed only in the Magellan Region (Fig. 8).

Of the 24 species recorded in the class Gastropoda, 25% (6 species) had a wide distribution, 4% (1 species) had a warm temperate southeastern Pacific-Magellanic distribution, and 46% (11 species) presented a Magellanic distribution, while 17% (4 species) presented a warm temperate southwestern Atlantic-Magellanic distribution and 8% (2 species) presented a Magellanic-Southern Ocean distribution (Fig. 8).

Finally, the class Bivalvia presented 56% of the species (5 species) with wide distribution, 11% presented a warm temperate southeastern Pacific-Magellanic distribution, Magellanic, warm temperate southwestern Atlantic-Magellanic, and Magellanic-Southern Ocean distribution, respectively (Fig. 8).

Shared species between sampling sites and the different biogeographic areas assessed showed variable values (Table 3). The highest ratio of similarity was observed in Bivalvia from Atlantic Patagonia (0.89), followed by Gastropoda in the same area (0.71). In third place are the Polyplacophora from the intermediate area of the South-eastern Pacific, Bivalvia from Uruguay and the Southern Ocean (0.56, respectively). However, lower values were observed in Gastropoda from Peru and Polyplacophora from Uruguay (0.00, which indicates no species shared with those areas).

**Table 3.** Zoogeographic affinities of molluscs recorded in this study: total species present for each area; number of shared species with this study; ratio of similarity and Simpson similarity coefficient. (1) from Ramirez et al. (2003), (2) Valdovinos (1999), (3) Carcelles (1950), (4) Scarabino (2003a), (5) Scarabino (2003b), (6) Scarabino (2004), (7) personal compilation, and (8) Griffiths et al. (2003).

		Total species	Shared species	Ratio of similarity	Simpson similarity coefficient
Perú (1)	Polyplacophora	33	3	0.33	0.091
	Gastropoda	543	0	0.00	0.000
	Bivalvia	357	1	0.11	0.003
Warm Temperate South-eastern Pacific (15°S–30°S), (2)	Polyplacophora	28	3	0.33	0.107
	Gastropoda	224	1	0.04	0.004
	Bivalvia	78	3	0.33	0.038
Intermediate area (30°S–40°S), (2)	Polyplacophora	29	5	0.56	0.172
	Gastropoda	239	5	0.21	0.021
	Bivalvia	84	4	0.44	0.048
Atlantic Patagonia *sensu lato* (36°S–52°S), (3)	Polyplacophora	23	4	0.44	0.174
	Gastropoda	156	17	0.71	0.109
	Bivalvia	57	8	0.89	0.140
Uruguay (4, 5, 6)	Polyplacophora	5	0	0.00	0.000
	Gastropoda	115	3	0.13	0.026
	Bivalvia	49	5	0.56	0.102
Antarctica (7)	Polyplacophora	6	3	0.33	0.500
	Gastropoda	337	7	0.29	0.021
	Bivalvia	224	4	0.44	0.018
Southern Ocean and adjacent areas (8)	Polyplacophora	–	–	–	–
	Gastropoda	500	8	0.33	0.016
	Bivalvia	287	5	0.56	0.017

The Simpson similarity coefficient showed the greatest value in Polyplacophora from Antarctica with 0.500 (Table 3). In second and third place are Poplyplacophora from the Atlantic Patagonia and intermediate area of South Eastern Pacific with 0.174 and 0.172, respectively (Table 3). Except for areas where there are no shared species, the lowest values were recorded in Bivalvia from Peru with 0.003, and Gastropoda from the Warm Temperate South-eastern Pacific with 0.004 (Table 3).

## Discussion

### Number and composition of species

The Magellan Region, defined in the database of Linse (1999) such as the Patagonian platform south of 41°S in the Pacific and Atlantic margins of South America, reports 381 marine species: 250 gastropods and 131 bivalves, not including polyplacophorans due to taxonomic problems with the group. Of the total species reported by Linse (1999), 278 inhabit depths less than 30 m, being considered “shallow-water species”: 180 gastropods and 98 bivalves. The 33 species recorded in this study correspond to 12% of the total shallow-water species cited: 13% for Gastropoda and 9% for Bivalvia.

Sirenko (2006) investigated the state of knowledge about the Polyplacophora from the Strait of Magellan and the Malvinas/Falkland Islands, recording a total of 17 species for the Strait of Magellan. However, the author was only able to collect 14 species, due to the rarity of some species, such as *Ischnochiton pusio*. Additionally, there are 11 other species of polyplacophorans cited for the Magellan Region, but Sirenko (2006) noted that these records were probably erroneous, given that these species are usually present in warmer waters. The 9 polyplacophoran species recorded in this study (2 Ischnochitonidae, 1 Callochitonidae, 4 Chitonidae, and 2 Mopaliidae) correspond to less than 47% of the species cited for the Strait of Magellan by Sirenko (2006).

Nevertheless, the percentages given above should be considered only as a reference, since some species could currently be considered junior synonyms of others following the publication of subsequent taxonomic revisions focused on specific groups (e.g. Pastorino 2005a, 2005b, Aranzamendi et al. 2009, Gonzalez-Wevar et al. 2010). Thus, the number of species varies, tending in some cases to decrease (e.g. Schwabe et al. 2006, Zelaya and Geiger 2007, Signorelli and Pastorino 2011). However, there have been descriptions of new species (e.g. Zelaya and Ituarte 2003, 2004), and a complete taxonomic overview is not possible at the time being.

The mollusc species richness recorded in this study represents a value over the average of those reported in other studies in the last 40 years in sublittoral environments in the Strait of Magellan (Table 4). Similarly, the study that presents the highest number of species (Aldea et al. 2011a) reported a total of 101 species of molluscs, but that study boarded a more extensive zone of the western micro-basin of the Strait of Magellan and some adjacent channels, where diverse substrates were studied. The present study is closer in quantity to the number of species carried out by Ríos et al. (2003), which was contained to the eastern micro-basin of the Strait of Magellan, recording 69 species between 30 and 50 meters (see Table 4). Projecting towards the fjord and canal zone in the Magellanic ecoregion, Dell (1971) reported 73 species in an extensive zone between 42°S and 55°S but did not consider the seafloor of the Strait of Magellan. Reid and Osorio (2000) recorded 62 species of molluscs in the sector of Estero Elefantes and Laguna San Rafael (46°S).

**Table 4.** Molluscs recorded in works since 1970 in the Strait of Magellan and adjacent channels. We took into account studies where sublittoral samples were collected.

Source	Latitude and depth	Gastropoda	Bivalvia	Polyplacophora	Total species†
Linse (2002)	52.9–53.7°S; 8–522m	17	1	0	18
Ríos et al. (2003)	52.6–52.8°S; 30–50m	38	21	10	69
Ríos et al. (2005)	52.3–53.9°S; 24–604m	8	6	1	15
Ríos et al. (2007)	53.0–53.6°S; ~8m	9	5	4	18
Thatje and Brown (2009)	52.3–55.2°S; 35–571m	5	15	1	21
Ríos et al. (2010)	52.3–52.5°S; ~16–~61m	1	3	0	4
Aldea et al. (2011a)	53.4–53.9°S; 5–20m	59	31	11	101
		20±8	12±4	4±2	35±13
**This record**	**53°S; ~10m**	**25**	**11**	**9**	**45**

† Morphospecies identified to generic level (“genus” sp.) are included.

From an ecological point of view, it is very difficult to carry out studies on communities and assemblages and be able to establish trophic groups, due to the lack of biologic studies about most of the mollusc species. For example, *Chiton bowenii* and *Nuttallochiton martiali* display unusual autecological aspects (Schwabe 2009). *Savatieria meridionale* should be compared with other species of the genus (Dell 1972), *Calliostoma nudum*, *Calliostoma modestulum* and *Photinastoma taeniatum* have a generic position that needs to be revised due to their similar characteristics (see Castellanos and Landoni 1989), *Pareuthria paessleri* and *Pareuthria janseni* have unknown developmental strategies (Pastorino and Penchaszadeh 2002). Thus, it is very important to conserve this type of environment, given that it shelters species that are considered by some authors to be “rare” or of low frequency (Dell 1971, Sirenko 2006, Rios et al. 2007). In this sense, algae beds of our sampling sites shelter ~38% of rare species for this habitat (see Table 1).

### Distribution aspects of the molluscs

Natural beds of *Gigartina skottsbergii* are characterized by a high species richness of molluscs. This study showed that the assemblage of molluscs that inhabit beds of *Gigartina skottsbergii* in the Strait of Magellan are species represented in the Magellanic Biogeographic Province, finding 38% of species that are exclusively distributed within this province. Gastropods in this study presented a high percentage of species with Magellanic distribution *sensu stricto* (Gastropoda = 45.8% and Bivalvia = 11.1%; see Fig. 8) contradicting Linse et al. (2006), who mentioned that for the Strait of Magellan, bivalves present a higher level of endemism than gastropods (Gastropoda = 13.3% and Bivalvia = 23.2%).

Other biogeographic studies carried out in the channels and fjords of Southern Chile showed that gastropods and bivalves have a higher similarity to molluscs from the Malvinas/Falkland Islands and South Georgia Islands (31% and 37%; Brandt et al. 1999). However, in our study, 74% of the species are present in the Malvinas/Falkland Islands and only 5 species (11%) are present in the South Georgia Islands. Therefore, the biogeographic study done by Zelaya (2005) for gastropods in the South Georgia Islands found that the affinity between the Magellanic Province and the South Georgia Islands is lower than those proposed by Brandt et al. (1999), finding only a 16% similarity with the Magellanic gastropods. Of the 24 species of gastropods recorded in this study, only *Iothia emarginuloides*, *Photinastoma taeniatum*, and probably *Margarella expansa* are reported for the South Georgia Islands. For that reason the affinity is quite low (13%).

In a complementary manner, upon comparison of the composition of the 16 genera of gastropods recorded in this study with those reported by Zelaya (2005) for the South Georgia Islands, only the genera *Iothia*, *Margarella*, *Photinastoma*, *Eatoniella*, and *Trophon* are present in both sites. In this manner, the low similarity can be observed between gastropod fauna recorded in this study and those from the South Georgia Islands. This low affinity between the Magellanic province and the South Georgia Islands not only occurs in molluscs, but data from other groups also supports the idea of including the South Georgia Islands within the Antarctic Region (De Broyer and Jazdewski 1993, Zelaya 2005, Spalding et al. 2007). The differences in the fauna composition can likely be explained by the difference in temperatures caused by the presence of the Antarctic convergence and the deep waters between the South Georgia Islands and the Magellanic Province (Zelaya 2005). However, in this study, only 42 species of molluscs were evaluated (corresponding to 12% of the shallow-water species from the Magellanic Province), and as a result, a larger number of samples and studies in different sectors of Magallanes could give better comparative information about the distribution of different mollusc species.

It is important to note that none of the biogeographic studies mentioned (Brandt et al. 1999, Linse et al. 2006) included the class Polyplacophora in their analysis. In this study, of the 9 species identified, 4 (44%) had a Magellanic distribution and highest similarity with Antarctica (see Table 3). Thus, it would be important to consider this group in future biogeographic research to better understand its current status.

Other biogeographic studies carried out in the Eastern Ocean of South America have demonstrated that the highest rates of endemism are found at high latitudes, principally in the Magellanic and Scotia Sea provinces (Fortes and Absalao 2011). At the same time, Fortes and Absalao (2011) mentioned that these high rates of endemism present in the Scotia Sea could be explained by the influence on the degree of isolation that the Antarctic creates over communities of this region (Clarke et al. 2004).

In general, the assemblage of molluscs recorded in this study showed low affinity with other provinces or regions in South America (see Table 3), and the largest proportion of similarity was presented in molluscs of Atlantic Patagonia and in the intermediate area of the Pacific (see Table 3), while the Simpson similarity coefficient in general presented low values, except for the Antarctic Polyplacophora. These results are important from the point of view of conservation of these benthic Magellanic ecosystems, given that an overexploitation of natural habitats of *Gigartina skottsbergii* would affect mostly endemic species of the Magellanic biogeographic province as well as other species distributed towards the Atlantic Patagonia and the intermediate area of the Pacific.

## Supplementary Material

XML Treatment for Ischnochiton stramineus

XML Treatment for Ischnochiton pusio

XML Treatment for Callochiton puniceus

XML Treatment for Tonicia lebruni

XML Treatment for Tonicia chilensis

XML Treatment for Tonicia atrata

XML Treatment for Chiton bowenii

XML Treatment for Plaxiphora aurata

XML Treatment for Nuttallochiton martiali

XML Treatment for Nacella deaurata

XML Treatment for Nacella flammea

XML Treatment for Nacella mytilina

XML Treatment for Iothia emarginuloides

XML Treatment for Fissurella picta picta

XML Treatment for Fissurella oriens

XML Treatment for Margarella violacea

XML Treatment for Margarella expansa

XML Treatment for Calliostoma nudum

XML Treatment for Calliostoma modestulum

XML Treatment for Photinastoma taeniatum

XML Treatment for Trochita pileus

XML Treatment for Fusitriton magellanicus

XML Treatment for Eatoniella nigra

XML Treatment for Eumetula pulla

XML Treatment for Savatieria meridionalis

XML Treatment for Pareuthria cerealis

XML Treatment for Pareuthria plumbea

XML Treatment for Pareuthria paessleri

XML Treatment for Pareuthria janseni

XML Treatment for Trophon geversianus

XML Treatment for Fuegotrophon pallidus

XML Treatment for Xymenopsis muriciformis

XML Treatment for Acteon biplicatus

XML Treatment for Aulacomya atra

XML Treatment for Mytilus edulis platensis

XML Treatment for Astarte longirostra

XML Treatment for Limea pygmaea

XML Treatment for Zygochlamys patagonica

XML Treatment for Austrochlamys natans

XML Treatment for Carditella naviformis

XML Treatment for Tawera elliptica

XML Treatment for Gaimardia trapesina

## Supplementary materials

Supplementary material 1 Appendix Data type: species list. Explanation note: Species excluded from the analysis of Table 3 (biogeographic analysis) This dataset is made available under the Open Database License (http://opendatacommons.org/licenses/odbl/1.0/). The Open Database License (ODbL) is a license agreement intended to allow users to freely share, modify, and use this Dataset while maintaining this same freedom for others, provided that the original source and author(s) are credited.Sebastián Rosenfeld, Cristian Aldea, Andrés Mansilla, Johanna Marambio, Jaime Ojeda
