# Supplementary material for: Richness, systematics, and distribution of molluscs associated with the macroalga Gigartina skottsbergii in the Strait of Magellan, Chile: A biogeographic affinity study
Source: Zookeys. 2015 Aug 31;(519):49–100. doi: 10.3897/zookeys.519.9676 (PMC4591604; doi:10.3897/zookeys.519.9676)
Supplement: Supplementary material 1 — Appendix [file zookeys-519-049-s001.doc]

**APPENDIX**

**Species excluded from the analysis of Table 3 (biogeographic analysis)**

**From Carcelles (1950): Patagonia**

Gastropoda

1. Tugalia antarctica (=Parmaphoridea melvilli)
2. Capulus compressus
3. Lamellaria ampla
4. Macron wrighti
5. Antistreptus magellanicus
6. Fusus acanthodes (=Trophon acanthodes)
7. Adelomelon beckii
8. Admete schythei
9. Admete carinata
10. Volvarina warreni (=V. patagonica)
11. Volvarina dozei
12. Spirotropis patagonica (=Drillia patagonica)
13. Drillia janseni
14. Drillia kophameli
15. Drillia suxdorfi
16. Agladrillia fuegiensis
17. Aforia goniodes (=Surcula clara)
18. Typhlodaphne purissima (=Mangelia)
19. Mangelia hyemalis
20. Mangelia fulvicans
21. Mangelia angustiplicata
22. Mangelia paessleri
23. Toledonia bullata (=Acteon)

Bivalvia

1. Nucula crenulata
2. Tindaria striata
3. Silicula fragilis
4. Acharax patagonicus (=S. macrodactyla)
5. Limopsis tenella
6. Philobrya atlantica
7. Acesta patagonica
8. Parathyasira magellanica
9. Microcardium delicatulum (=Cardium)
10. Pandora braziliensis (=Kennerlya)
11. Policordia radiata
12. Cuspidaria simillina

**From Valdovinos (1999): Warm Temperate Southeastern Pacific - 5°S-30°S**

Gastropoda

1. Diodora codoceae
2. Bathybembix humboldti
3. Bathybembix macdonaldi
4. Calliostoma chilena
5. Calliostoma delli
6. Janthina exigua (pelágica)
7. Capulus ungaricuides
8. Polinices constrictus
9. Polinices crawfordianus
10. Trophon bahamondei
11. Columbarium tomicici
12. Aeneator fontainei
13. Aeneator castillai
14. Miomelon alarconi
15. Cancellaria stuardoi
16. Ptychosyrinx chilensis
17. Scaphander interruptus
18. Limacina trochiformis (pelágica)
19. Pneumoderma boasi (=violaceum) (pelágica)
20. Dexiobranchaea polycotyla (pelágica)
21. Dexiobranchaea simplex (pelágica)
22. Phyllinoe bucephala (pelágica)
23. Fiona pinnata (pelágica)

Bivalvia

1. Ennucula grayi
2. Ennucula puelcha
3. Nuculana cuneata
4. Lucinoma aequizonata
5. Mysella deanneae
6. Mysella mabillei
7. Montacutona montemarensis (=Phytinella)
8. Calyptogena australis
9. Xylophaga globosa
10. Poromya mactroides
11. Cuspidaria patagonica

**From Valdovinos (1999): Intermediate area - 30°S-40°S**

Gastropoda

1. Diodora codoceae
2. Lucapina maullina
3. Bathybembix humboldti
4. Bathybembix macdonaldi
5. Calliostoma chilena
6. Calliostoma delli
7. Janthina exigua (pelágica)
8. Capulus ungaricuides
9. Polinices crawfordianus
10. Marseniopsis pacifica
11. Columbarium tomicici
12. Aeneator fontainei
13. Ptychosyrinx chilensis
14. Mangelia paessleri
15. Scaphander interruptus

Bivalvia

1. Ennucula grayi
2. Ennucula puelcha
3. Nuculana cuneata
4. Yoldiella chilenica
5. Tindaria virens
6. Acesta patagonica
7. Lucinoma aequizonata
8. Mysella deanneae
9. Mysella mabillei
10. Calyptogena australis
11. Xylophaga globosa
12. Microcardium delicatulum (=Trachycardium)
13. Poromya mactroides
14. Cuspidaria patagonica

**From Ramírez (2003; *sensu* Alamo & Valdivieso 1997): Perú**

Polyplacophora

1. Leptochiton opacus

Gastropoda

1. Bathybembix (Bathybembix) bairdii
2. Bathybembix (Bathybembix) humboldti
3. Bathybembix (Bathybembix) macdonaldi
4. Bathybembix (Solaricida) equatorialis
5. Calliostoma aequiscultum
6. Calliostoma (Otukaia) chilena
7. Bathysciadium pacificum
8. Architectonica (Discotectonica) placentalis
9. Strombus (Lentigo) granulatus
10. Amaea (Scalina) tehuanarum
11. Janthina janthina
12. Crepidula striolata
13. Crucibulum (Crucibulum) monticulus
14. Natica (Natica) scethra
15. Polinices agujanus
16. Oocorys elevata
17. Pteropurpura (Centrifuga) centrifuga
18. Phos (Metaphos) articulatus
19. Nassarius catallus
20. Adelomelon benthalis
21. Calliotectum vernicosum
22. Aforia goodei
23. Anticlinura peruviana
24. ?Leucosyrix clionella
25. Xanthodaphne egregia
26. Scaphander cylindrellus

Bivalvia

1. Acharax johnsoni
2. Nucula (Leionucula) colombiana
3. Nucula agujana
4. Nucula (Nucula) chrysocome
5. ?Nucula savatieri
6. Malletia (Malletia) peruviana
7. Nuculana (Jupiteria) callimene
8. Spinula (Spinula) calcar
9. Limopsis (Limopsis) zonalis
10. Chione (Lirophora) kellettii
11. Macoma (Macoploma) medioamericana
12. Cuspidaria (Cuspidaria) chilensis
13. ?Cuspidaria (Cuspidaria) patagonica

**From Scarabino (2003b): Uruguay**

Bivalvia

1. Deminucula atecellana
2. Ennucula perforata
3. Pristigloma alba
4. Pristigloma nitens
5. Propeleda carpentieri
6. Thestyleda lousiae
7. Ledella aberrata
8. Ledella jamesi
9. Ledella pustulosa
10. Ledella sublevis
11. Ledella ultima
12. Bathyspinula hilleri
13. Bathyspinula scheltemai
14. Yoldiella americana
15. Yoldiella argentinensis
16. Yoldiella biguttata
17. Yoldiella blanda
18. Yoldiella curta
19. Yoldiella extensa
20. Yoldiella fabula
21. Yoldiella inconspicua
22. Yoldiella jeffreysi
23. Yoldiella robusta
24. Yoldiella similiris
25. Tindaria callistiformis
26. Neilonella whoi
27. Austrotindaria championi
28. Clencharia abyssorum
29. Katadesmia cuneata
30. Prelametila clarkei
31. Silicula fragilis
32. Silicula mcalesteri
33. Dacrydium ockelmenni
34. Limopsis spicata
35. Cuspidaria barnardi
36. Cuspidaria circinata
37. Cuspidaria parva
38. Cuspidaria platensis
39. Cardiomya knudseni
40. Bathyneaera hadalis
41. Myonera alleni
42. Myonera paucistriata
43. Protocuspidaria verityi
44. Verticordia quadrata
45. Lyonsiella fragilis
46. Lyonsiella perplexa
47. Policordia gemma
48. Mysella verrilli
49. Thyasira succisa
50. Thyasira equalis
51. Thyasira subequatoria
52. Thysaria subovata
53. Axinulus brevis
54. Axinulus croulinensis
55. Mendicula carrozae
56. Mendicula inflata
57. Mendicula ferruginosa
58. Mendicula transversa
59. Vesicomya atlantica
60. Isorropodon elongatum
61. Neolepton profundorum

**From Scarabino (2004): Uruguay**

Gastropoda

1. Asthelys simplex
2. Brookula powelli
3. Litiopa melanostoma (pelágica)
4. Rissoa herwigia
5. Atlanta fusca (pelágica)
6. Atlanta peronii (pelágica)
7. Oxygyrus keraudreni (pelágica)
8. Firoloida desmarestia (pelágica)
9. Cardiapoda richardi (pelágica)
10. Ranella olearium
11. Janthina janthina (pelágica)
12. Recluzia rollandiana (pelágica)
13. Coronium coronatum
14. Americominella duartei
15. Aforia gonoides
16. Stylocheilus citrinus (pelágica)
17. Limacina bulimoides (pelágica)
18. Limacina helicina (pelágica)
19. Limacina inflata (pelágica)
20. Limacina lesuerii (pelágica)
21. Limacina retroversa (pelágica)
22. Limacina trochiformis (pelágica)
23. Cavolinia gibbosa (pelágica)
24. Cavilonia inflexa (pelágica)
25. Cavilonia uncinata (pelágica)
26. Diacavilonia longirostris (pelágica)
27. Diacria trispinosa (pelágica)
28. Clio cuspidata (pelágica)
29. Clio pyramidata (pelágica)
30. Creseis acicula (pelágica)
31. Creseis vírgula (pelágica)
32. Styliola subula (pelágica)
33. Cueviernia columnella (pelágica)
34. Cymbulia peroni (pelágica)
35. Clione antárctica (pelágica)
36. Philine thurmanni
37. Glaucus atlanticus
38. Fionna pinnata

**Antárctica (Personal compilation)**

Polyplacophora

1. Stenosemus exaratus
2. Stenosemus simplicissimus
3. Callochiton gaussae
4. Callochiton steinenii
5. Leloupia belgicae
6. Nuttallochiton mirandus
7. Nuttallochiton hyadesi
8. Hemiarthrum setulosum

Gastropoda

1. Lacuna abyssicola
2. Lusitromina abyssorum
3. Margarella achilles
4. Amphissa acuminata
5. Puncturella agulhasae
6. Trenchia agulhasae
7. Eatoniella ainsworthi
8. Guivillea alabastrina
9. Philine alata
10. Eatoniella alboelata
11. Falsimohnia albozonata
12. Calliostoma alertae
13. Onoba algida
14. Proneptunea amabilis
15. Melanella amblia
16. Prosipho amiantus
17. Thieleella amoena
18. Lamellaria ampla
19. Solariella anarensis
20. Calliostoma anderssoni
21. Diaphana anderssoni
22. Frovina angularis
23. Probuccinum angulatum
24. Torellia angulifera
25. Pleurotomella annulata
26. Nothoadmete antarctica
27. Torellia antarctica
28. Cerithiopsilla antarctica
29. Newnesia antarctica
30. Acirsa antarctica
31. Murdochella antarctica
32. Melanella antarctica
33. Cornisepta antarctica
34. Parmaphoridea antarctica
35. Trachysma antarctica
36. Retusa antarctica
37. Seguenzia antarctica
38. Brookula antarctica
39. Trochaclis antarctica
40. Calliotropis antarctica
41. Solariella antarctica
42. Conorbela antarctica
43. Cochliolepis antarctica
44. Acteon antarcticus
45. Prosipho antarctidis
46. Philine apertissima
47. Pontiothauma archibenthale
48. Savatieria areolata
49. Trenchia argentinae
50. Paradmete arnaudi
51. Prosipho astrolabiensis
52. Cerithiella astrolabiensis
53. Omalogyra atomus atomus
54. Melanella aucklandica
55. Iredalina aurantia
56. Antarctoneptunea aurora
57. Prosipho aurora
58. Lamellariopsis aurora
59. Cerithiopsilla austrina
60. Banzarecolpus austrina
61. Pusillina averni
62. Leucosyrinx badenpowelli
63. Volutomitra banksi
64. Solariella bathyantarctica
65. Crenatosipho beaglensis
66. Falsitromina bella
67. Eatoniella bennetti
68. Antimargarita bentarti
69. Falsimargarita benthicola
70. Penion benthicolus
71. Bulbus benthicolus
72. Fulgurofusus benthocallis
73. Tropidomarga biangulata
74. Zerotula bicarinata
75. Parabuccinum bisculpta
76. Cerithiopsilla bisculpta
77. Scissurella bountyensis
78. Notoficula bouveti
79. Paradmete breidensis
80. Solariella brychius
81. Cerithiopsilla burdwoodianus
82. Micrelenchus caelatus caelatus
83. Micrelenchus caelatus mortenseni
84. Brookula calypso
85. Prosipho cancellatus
86. Brookula capensis
87. Admete carinata
88. Carenzia carinata
89. Prosipho certus
90. Solariella charopus charopus
91. Capulus chilensis
92. Pareuthria chlorotica
93. Chlanidota chordata
94. Prosipho chordatus
95. Cerithiopsilla cincta
96. Pisinna circumlabra
97. Scissurella clathrata
98. Lodderia coatsiana
99. Puncturella cognata
100. Capulus compressus
101. Prosipho congenitus
102. Puncturella conica
103. Marseniopsis conica
104. Prosipho contrarius
105. Melanella convexa
106. Savatieria coppingeri
107. Torellia cornea
108. Zerotula coronata
109. Typhlodaphne corpulenta
110. Mangelia costata
111. Probuccinum costatum
112. Trophon coulmanensis
113. Trophon coulmanensis multilamellatus
114. Liotella crassicostata
115. Prosipho crassicostatus
116. Paradmete crymochara
117. Cylichna cumberlandiana
118. Paradmete curta
119. Trophon cuspidarioides
120. Fictonoba cymatodes
121. Prosipho daphnelloides
122. Lorabela davisi
123. Brookula decussata
124. Ringicula delecta
125. Nothoadmete delicatula
126. Triphora delicatula
127. Bathybembix delicatula
128. Chlanidota densesculpta
129. Cirsonella densilirata
130. Comptella devia
131. Ancillaria dimidiata
132. Tromina dispectata
133. Bathybembix drakei
134. Trophon drygalskii
135. Volvarina ealesae
136. Trophon echinolamellatus
137. Neactaeonina edentula
138. Skenella edwardiensis
139. Lamellaria elata
140. Seguenzia elegans
141. Prosipho elongatus
142. Parabuccinum eltanini
143. Asperiscala eltanini
144. Falsilunatia eltanini
145. Calliotropis eltanini
146. Miomelon eltanini
147. Pleurotomella endeavourensis
148. Admete enderbyensis
149. Fissurisepta enderbyensis
150. Trophon enderbyensis
151. Pleurotomella enderbyensis
152. Liotella endevourensis
153. Mesoginella ergastula
154. Seguenzia eritima
155. Torellia exilis
156. Patelloida exilis
157. Maoricrater explorata
158. Melanella exulata
159. Scissurella fairchildi
160. Typhlomangelia fluctuosa
161. Merelina foliata
162. Neactaeonina fragilis
163. Paradmete fragillima
164. Retusa frigida
165. Pleurotomella frigida
166. Banzarecolpus frigida
167. Pareuthria fuscata
168. Prosipho fuscus
169. Meteuthria futilis
170. Belaturricula gaini
171. Munditia gaudens
172. Prosipho gaussianus
173. Cylichna gelida
174. Falsimargarita gemma
175. Cerithiopsilla georgiana
176. Skenella georgiana
177. Cylichna georgiana
178. Falsimargarita georgiana
179. Tractolira germonae
180. Philine gibba
181. Prosipho glacialis
182. Bela glacialis
183. Aforia gonoides
184. Prosipho gracilis
185. Admete haini
186. Skenella hallae
187. Prosipho hedleyi
188. Pontiothauma hedleyi
189. Zerotula hedleyi
190. Trilirata herosae
191. Intortia homocostata
192. Prosipho hunteri
193. Volvarina hyalina
194. Trachysma ignobile
195. Submargarita impervia
196. Zerotula incognita
197. Hemiaclis incolorata
198. Radiacmea inconspicua
199. Frovina indecora
200. Diaphana inflata
201. Calliotropis infundibulum
202. Leptocollonia innocens
203. Typhlodaphne innocentia
204. Torellia insignis
205. Chlanidota invenusta
206. Prosipho iodes
207. Sinezona iota
208. Falsimargarita iris
209. Fusinella jucunda
210. Sassia kampyla
211. Splendrillia kapuranga
212. Solariella kempi
213. Philine kerguelensis
214. Cirsonella kerguelensis
215. Claviscala kuroharai
216. Lepsithais lacunosus
217. Sinezona laevigata
218. Turbonilla lamyi
219. Torellia lanata
220. Sinezona laqueus
221. Calliotropis lateumbilicata
222. Turritellopsis latior
223. Aforia lepta
224. Turbonilla lillingtoniana
225. Lissotesta liratula
226. Zalipais lissa
227. Ancillaria longispira
228. Seguenzia louiseae
229. Dentimargo lurida
230. Sinezona lyallensis
231. Incisura lytteltonensis
232. Submargarita macknighti
233. Macquariella macphersonnae
234. Admete magellanica
235. Epitonium magellanicum
236. Antistreptus magellanicus
237. Aforia magnifica
238. Trophon malvinarum
239. Lissotesta mammillata
240. Antimargarita maoria
241. Prunum martini
242. Waipaoa marwicki
243. Parmaphoridea mawsoni
244. Leucosyrinx mawsoni
245. Calliostoma megaloprepes
246. Litiopa melanostoma
247. Parmaphoridea melvilli
248. Niveria memorata
249. Onoba merelinoides
250. Archiminolia meridiana
251. Munditia meridionalis
252. Sinuber microstriatum
253. Pisinna minor
254. Lissotesta minutissima
255. Cominella mirabilis
256. Torellia mirabilis
257. Perissodonta mirabilis
258. Iredalina mirabilis
259. Cominella mirabilis nuptialis
260. Chlanidotella modesta
261. Marseniopsis mollis
262. Zeatrophon mortenseni mortenseni
263. Aforia multispiralis
264. Prosipho mundus
265. Typhlodaphne nipri
266. Gadinalea nivea
267. Puncturella noachina
268. Calliostoma nordenskjoldi
269. Rissoella notabilis
270. Submargarita notalis
271. Lorabela notophila
272. Falsilunatia notorcadensis
273. Baryspira novaezealandica benthicola
274. Sigapatella novaezelandiae
275. Zerotula nummaria
276. Benhamina obliquata
277. Bathydomus obtectus
278. Trichosirius octocarinatus
279. Antarctodomus okutanii
280. Melarhaphe oliveri
281. Eumetula ornata
282. Retusa pachys
283. Marseniopsis pacifica
284. Chlanidota palliata
285. Skenella paludinoides
286. Pleurotomella papyracea
287. Leucosyrinx paratenoceras
288. Chlanidota paucispiralis
289. Columbella paxillus
290. Prosipho pellitus
291. Propilidium pelseneeri
292. Calliotropis pelseneeri pelseneeri
293. Calliotropis pelseneeri rossiana
294. Lorabela pelseneri
295. Paradmete percarinata
296. Asterophila perknasteri
297. Buccinulum pertinax
298. Scissurella petermannensis
299. Diaphana pfefferi
300. Brookula pfefferi
301. Merelina plaga
302. Torellia planispira
303. Typhlodaphne platamodes
304. Lorabela plicatula
305. Streptocionella pluralis
306. Parabuccinum polyspeira
307. Volutomitra porcellana
308. Lepetella postapicula
309. Antarctodomus powelli
310. Falsitromina powelli
311. Rissoella powelli
312. Brookula powelli
313. Antimargarita powelli
314. Natica prasina
315. Prosipho priestleyi
316. Typhlomangelia principalis
317. Lophiotoma pseudoannulata
318. Provocator pulcher
319. Calliostoma punctulatum urbanior
320. Typhlodaphne purissima
321. Prosipho pusillus
322. Parabuccinum rauscherti
323. Aeneator recens
324. Pisinna rekohuana
325. Micropleurotoma remota
326. Prosipho reversa
327. Mathilda rhigomaches
328. Rissoella rissoaformis
329. Incisura rosea
330. Pleurotomella rossi
331. Proneptunea rossiana
332. Pseudamauropsis rossiana
333. Brookula rossiana
334. Liotella rotula
335. Proneptunea rufa
336. Odostomia rugata rugata
337. Trophon scolopax
338. Miomelon scoresbyana
339. Venustatrochus secundus
340. Minolia semireticulata
341. Pellilitorina setosa
342. Bathydomus setosus
343. Cerithiella seymouriana
344. Prosipho shiraseae
345. Pickenia signyensis
346. Prosipho similis
347. Cerithiella similis
348. Pleurotomella simillima
349. Falsitromina simplex
350. Skenella sinapi
351. Torellia smithi
352. Antimargarita smithiana
353. Marseniopsis soliditesta
354. Melanella solitaria
355. Frovina soror
356. Calliostoma spectabile
357. Admete specularis
358. Marseniopsis spherica
359. Prosipho spiralis
360. Zerotula stellapolaris
361. Dentimargo stewartiana
362. Buccinulum strebeli
363. Lissotesta strebeli
364. Buccinulum strebeli exsculptum
365. Emarginula striatula
366. Belalora striatula
367. Submargarita studeri
368. Spirotropis studeriana
369. Melanella subantarctica
370. Arielia subantarctica
371. Capulus subcompressus
372. Proneptunea subfenestra
373. Pisinna subfusca
374. Cerithiella superba
375. Scissurella supraplicata
376. Acirsa symphylla
377. Marseniopsis syowaensis
378. Omalogyra taludana
379. Parvaplustrum tenerum
380. Probuccinum tenerum
381. Rugulina tenuis
382. Antarctodomus thielei
383. Falsimargarita thielei
384. Paxula transitans
385. Typhlodaphne translucida
386. Lusitromina traverseensis
387. Zerotula triangulata
388. Falsitromina tricarinata
389. Cingula trifasciata
390. Galeodea triganceae
391. Aforia trilix
392. Trilirata triregis
393. Retusa truncata
394. Prosipho tuberculatus
395. Melanella tumidula
396. Miomelon turnerae
397. Prosipho turrita
398. Belaturricula turrita multispiralis
399. Belaturricula turrita turrita
400. Paradmete typica
401. Skenella umbilicata
402. Submargarita unifilosa
403. Microdiscula vanhoeffeni
404. Chlanidota vestita
405. Lacuna vincta
406. Uberella vitrea
407. Skenella wareni

Bivalvia

1. Malletia abyssorum
2. Poromya adelaidis
3. Dacrydium albidum
4. Lucinoma antarctica
5. Tindaria antarctica
6. Yoldiella antarctica
7. Lyonsiella aotearoa
8. Lyonsia arcaeformis
9. Nucula austrobenthalis
10. Pronucula benguelana
11. Nuculana bicuspidata
12. Arthritica bifurca
13. Pronucula bollonsi
14. Thyasira bongraini
15. Pandora braziliensis
16. Philobrya capillata
17. Pseudokellya cardiformis
18. Pseudotindaria championi
19. Pandora cistula
20. Cuspidaria concentrica
21. Talochlamys consociata
22. Mesopeplum convexum
23. Condylocardia crassicosta
24. Parvithracia cuneata
25. Cuspidaria cuspidata
26. Bathyarca cybaea
27. Borniola decapitata
28. Tindaria diaphana
29. Lyonsia elegantula
30. Ennucula eltanini
31. Limopsis enderbyensis
32. Cuspidaria fairchildi
33. Myonera fragilissima
34. Entodesma fretalis
35. Cyclochlamys gaussianus
36. Pseudokellya georgiana
37. Ennucula georgiana
38. Malletia gigantea
39. Tellina gilchristi
40. Xylophaga globosa
41. Escalima goughensis
42. Pseudokellya gradata
43. Ennucula grayi
44. Cyclochlamys hexagonalis
45. Limopsis hirtella
46. Moerella huttoni
47. Yoldiella indolens
48. Pseudokellya inexpectata
49. Cuspidaria infelix
50. Malletia johnsoni
51. Cuspidaria kerguelensis
52. Limopsis knudseni
53. Adacnarca limopsoides
54. Propeleda longicaudata
55. Limopsis longipilosa
56. Limopsis mabilliana
57. Lyonsia malvinensis
58. Propeamussium meridionale
59. Thracia meridionalis
60. Pronucula mesembrina
61. Spinula messanensis
62. Poromya microsculpta
63. Cuspidaria minima
64. Pachykellya minima
65. Sheldonella minutalis
66. Cuspidaria morelandi
67. Talochlamys multicolor
68. Cuspidaria multicostata
69. Talochlamys multistriata
70. Modiolus neozelandicus
71. Nucula nitidula
72. Benthocardiella obliquata
73. Benthocardiella obliquata bountyensis
74. Yoldiella oblonga
75. Limatula ovalis
76. Malletia pallida
77. Dacrydium panamensis
78. Melliteryx parva
79. Silicula patagonica
80. Solemya patagonica
81. Malletia pellucida
82. Philobrya pinctata
83. Lissarca pisum
84. Yoldiella profundorum
85. Hyalopecten pudicus
86. Venericardia purpurata
87. Policordia radiata
88. Leptomya retiaria
89. Leptomya retiaria auklandica
90. Nucula rossiana
91. Pachykellya rotunda
92. Silicula rouchi
93. Ptychocardia rudis
94. Yoldiella sabrina
95. Limopsis scabra
96. Limopsis scotiana
97. Kellia simulans
98. Bathyarca sinuata
99. Bathyarca strebeli
100. Tindaria striata
101. Benthocardiella striatula
102. Lucina subfragilis
103. Kellia suborbicularis
104. Limatula suteri
105. Parvithracia suteri
106. Lepidocardia tellenoidea
107. Cuspidaria tenella
108. Limopsis tenella
109. Limopsis tenella tenella
110. Pronucula tenuis
111. Poromya tornata
112. Verticipronus tristanenis
113. Cuspidaria undata
114. Poromya undosa
115. Yoldiella valettei
116. Ptychocardia vanhoeffeni
117. Tindaria virens

**From Linse (1999): Magellanic Province**

Gastropoda

1. Parmaphoridea antarctica (Strebel, 1907)
2. Parmaphoridea melvilli (Thiele, 1912)
3. Puncturella conica (d'Orbigny, 1841)
4. Anatoma conica (d'Orbigny, 1841)
5. Calliostoma moebiusi Strebel, 1905
6. Calliostoma modestulum Strebel, 1908
7. Calliostoma nordenskjoldi Strebel, 1908
8. Calliostoma nudiusculum (Martens, 1881)
9. Cerithiopsilla burdwoodiana (Melvill & Standen, 1912)
10. Colpospirella algida (Melvill & Standen, 1912)
11. Mathilda argentina Castellanos, 1990
12. Papuliscala diminuta Castellanos, Rolán & Bartolotta, 1987
13. Melanella salvadori Castellanos, Rolán & Bartolotta, 1987
14. Leiostraca carforti Rochebrune & Mabille, 1889
15. Fartulum magellanicum di Geronimo, Privitera & Valdovinos, 1995
16. Capulus chilensis Dall, 1904
17. Capulus compressus E. A. Smith, 1891
18. Marseniopsis pacifica Bergh, 1886
19. Lamellaria ampla Strebel, 1906
20. Lamellaria elata Strebel, 1906
21. Aforia goniodes (Watson, 1881)
22. Belalora cunninghami (E. A. Smith, 1881)
23. Drillia suxdorfi Strebel, 1905
24. Drillia janseni Strebel, 1905
25. Spirotropis patagonica (d'Orbigny, 1841)
26. Agladrillia fuegiensis (E. A. Smith, 1888)
27. Leucosyrinx angusteplicata (Strebel, 1905)
28. Leucosyrinx taludana Castellanos & Landoni, 1993
29. Propebela profunda Castellanos & Landoni, 1993
30. Typhlodaphne purissima (Strebel, 1908)
31. Typhlodaphne strebeli A. W. B. Powell, 1951
32. Amphissa acuminata (E. A. Smith, 1915)
33. Amphissa cancellata (Castellanos, 1979)
34. Anomacme smithi (Strebel, 1905)
35. Antistreptus rolani Castellanos, 1986
36. Antistreptus magellanicus Dall, 1902
37. Parabuccinum bisculptum (Dell, 1990)
38. Parabuccinum eltanini (Dell, 1990)
39. Parabuccinum polyspeira (Dell, 1990)
40. Parabuccinum rauscherti Harasewych, Kantor & Linse, 2000
41. Falsitromina bella (A. W. B. Powell, 1951)
42. Falsitromina fenestrata (A. W. B. Powell, 1951)
43. Falsitromina powelli Dell, 1990
44. Falsitromina simplex (A. W. B. Powell, 1951)
45. Meteuthria multituberculata (Castellanos, Rolán & Bartolotta, 1987)
46. Parficulina problematica (A. W. B. Powell, 1951)
47. Prosipho antarctidis (Pelseneer, 1903)
48. Savatieria chordata Castellanos, Rolán & Bartolotta, 1987
49. Adelomelon beckii (Broderip, 1836)
50. Miomelon eltanini Dell, 1990
51. Miomelon scoresbyanum A. W. B. Powell, 1951
52. Miomelon turnerae Dell, 1990
53. Odontocymbiola canigiai Vazquez & Caldini, 1992
54. Odontocymbiola pescalia Clench & Turner, 1964
55. Odontocymbiola subnodosa (Leach, 1814)
56. Volvarina dozei (Rochebrune & Mabille, 1889)
57. Volvarina warreni (Marrat, 1876)
58. Trophon triacanthus Castellanos, Rolán & Bartolotta, 1987
59. Xymenopsis buccinea (Lamarck, 1816)
60. Eulimella xenophyes (Melvill & Standen, 1912)
61. Odostomia nova Castellanos, 1982
62. Acteon elongatus Castellanos, Rolán & Bartolotta, 1987
63. Toledonia punctata Thiele, 1912
64. Cylichna cumberlandiana (Strebel, 1908)
65. Cylichna georgiana (Strebel, 1908)
66. Philine falklandica A. W. B. Powell, 1951
67. Limacina helicina helicina (Phipps, 1774)
68. Limacina retroversa retroversa (Fleming, 1823)
69. Thilea procera Strebel, 1908
70. Clione antarctica E. A. Smith, 1902

Bivalvia

1. Nucula falklandica Preston, 1912
2. Nucula exigua Sowerby, 1833
3. Ennucula eltanini Dell, 1990
4. Yoldiella chilenica (Dall, 1908)
5. Yoldiella granula (Dall, 1908)
6. Yoldia (Yoldiella) infrequens Dall, 1908
7. Malletia inequalis Dall, 1908
8. Tindaria virens (Dall, 1890)
9. Propeleda longicaudata (Thiele, 1912)
10. Silicula patagonica Dall, 1908
11. Solemya macrodactyla Mabille & Rochebrune, 1889
12. Limopsis hirtella Mabille & Rochebrune, 1889
13. Limopsis tenella dalli Lamy, 1912
14. Dacrydium panamensis Knudsen, 1970
15. Cyclopecten subhyalinus (E. A. Smith, 1885)
16. Cyclopecten falklandicus Dell, 1964
17. Acesta patagonica (Dall, 1902)
18. Aligena pisum Da1l 1900
19. Waldo parasiticus (Dall, 1876)
20. Mysella sculpta Soot-Ryen, 1957
21. Neolepton falklandicum Dell, 1964
22. Neolepton umbonatum Smith, 1885
23. Cyclocardia compressa (Reeve, 1843)
24. Trachycardium delicatulum (E. A. Smith, 1915)
25. Pandora braziliensis Sowerby II, 1874
26. Entodesma fretalis (Dall, 1915)
27. Poromya adelaidis (Hedley, 1916)
28. Poromya chilensis Dall, 1908
29. Poromya mactroides Dall, 1889
30. Cuspidaria infelix Thiele, 1912
31. Cuspidaria tenella Smith, 1907
32. Cuspidaria chilensis Dall, 1890
33. Lyonsiella radiata Dall, 1889
